# Supplementary figures and images for: Characterization of a new type of neuronal 5-HT G- protein coupled receptor in the cestode nervous system
Source: PLoS One. 2021 Nov 11;16(11):e0259104. doi: 10.1371/journal.pone.0259104 (PMC8584985; doi:10.1371/journal.pone.0259104)

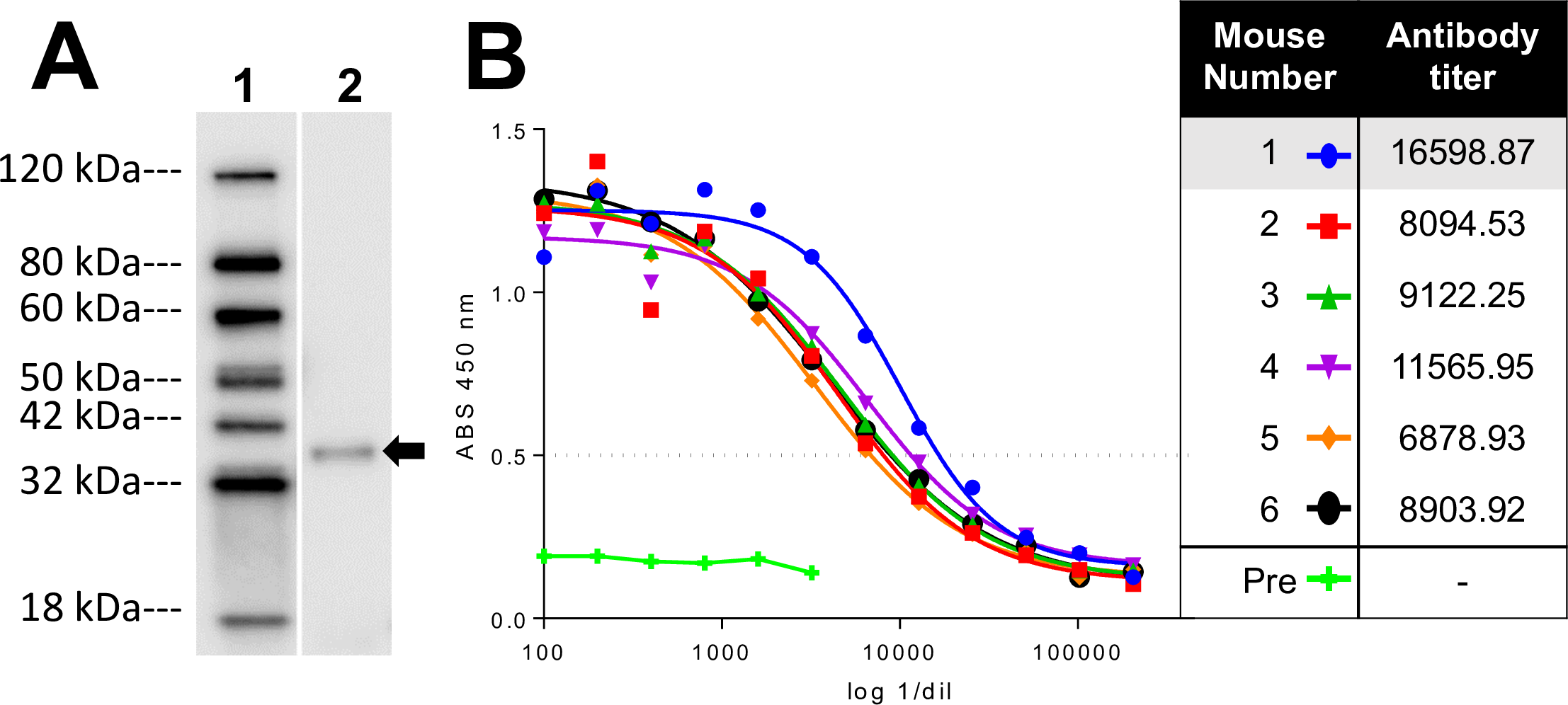

Supplement: S1 Fig — A) Lane 1, GenScript protein marker (Cat. No. M00521); lane 2, 5 μg of Eca-5-HT1aICL3. The antigen was run in polyacrylamide gel electrophoresis and transferred to a nitrocellulose membrane. The membrane was probed with antibody against poly-histidine tag. The arrow shows the position of the band detected. B) The recombinant antigen produced was administered in six mice and then anti-Eca-5-HT1aICL3 antibody titers were evaluated by ELISA technique. End-point titer was calculated as the reciprocal of the dilution with an Absorbance 450 nm = 0.5. The antibody titers are indicated in the table from the right. “Pre” represents the specific antibody titer of pre-immunized mice. (TIF) [file pone.0259104.s001.tif]

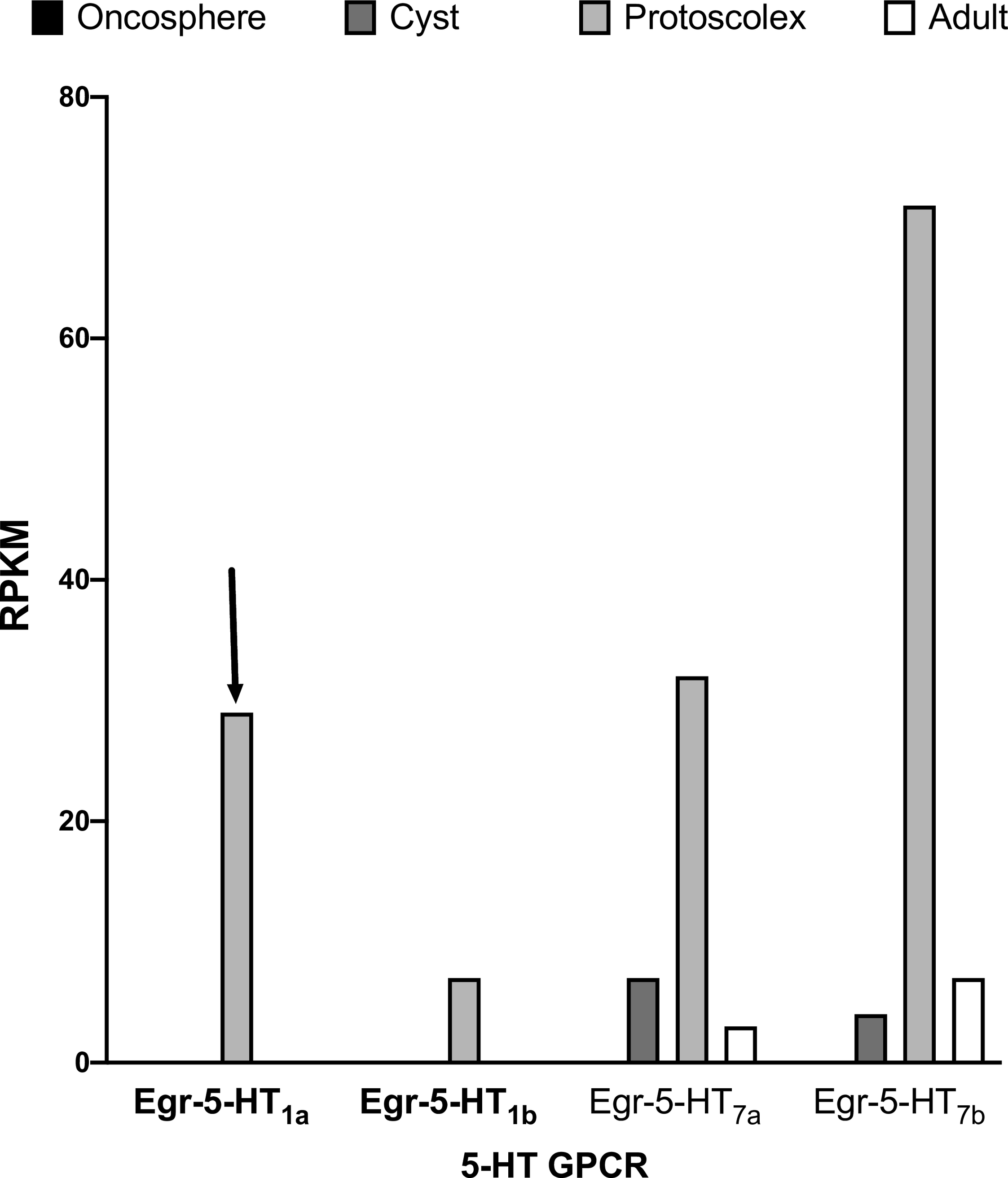

Supplement: S2 Fig — Egr-5-HT1a, Egr-5-HT1b, Egr-5-HT7a and Egr-5-HT7b GPCRs transcriptional expression levels are shown as RPKM (Reads Per Kilobase Million). Comparison of gene expression levels, determined by RNAseq, in several developmental stages of Echinococcus granulosus sensu stricto (G1): Oncosphere, Cyst, Protoscolex and Adult [26]. The closest orthologue of the receptor studied here, Egr-5-HT1a, was marked in bold and the level of transcript expression of this receptor in the protoscolex stage was marked with an arrow. With exception of the protoscolex larval stage, no transcript expression was observed for this receptor in other stages of the parasite. (TIF) [file pone.0259104.s002.tif]

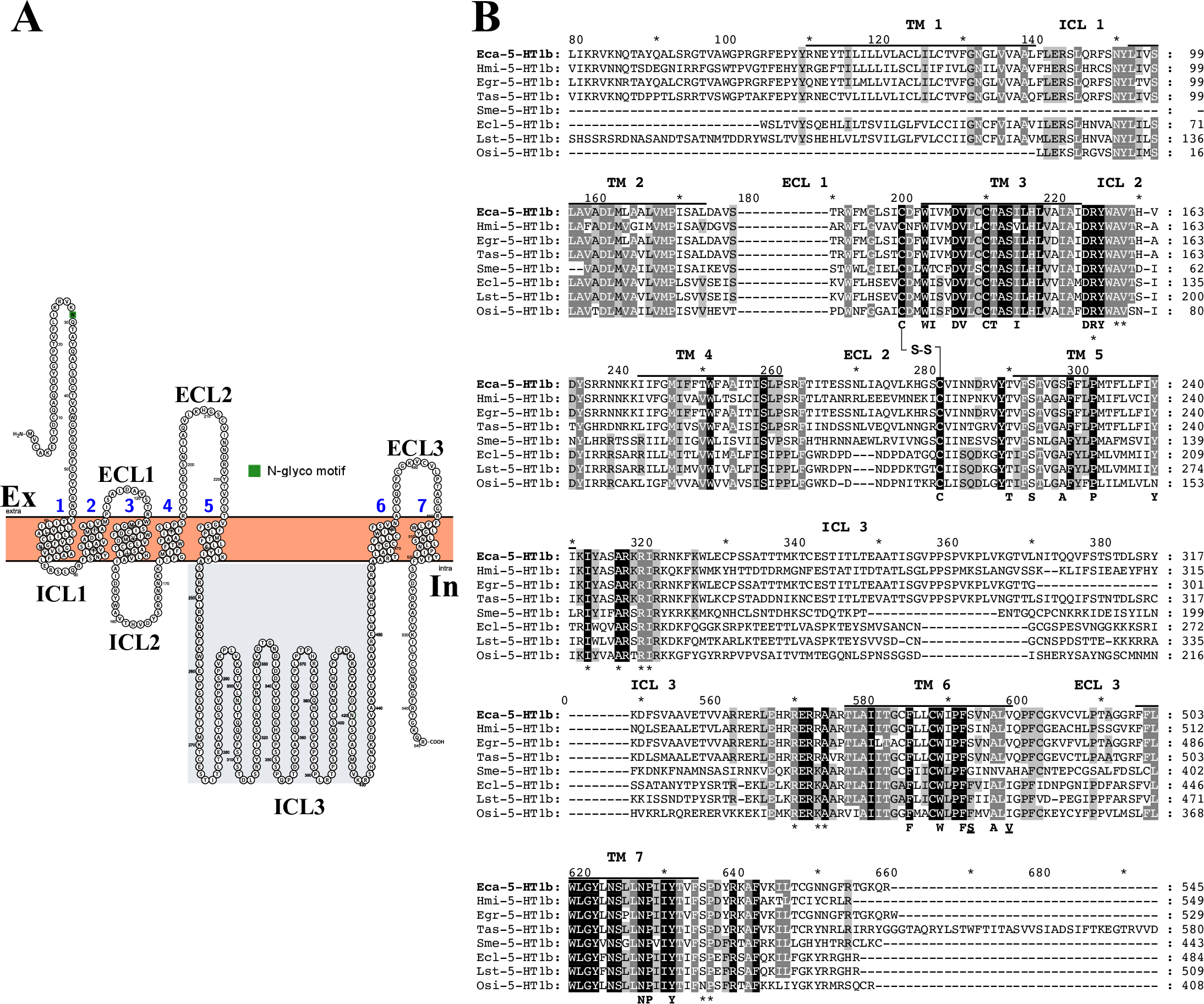

Supplement: S3 Fig — A. The bidimensional structure representation and prediction of residues of potential N-glycosylation of Eca-5-HT1b were obtained with the Protter program (http://www.enzim.hu/hmmtop/index.php). The intracellular and extracellular loops are indicated as ICL and ECL respectively. The third intracellular loop used for antibody generation is shaded in grey. Residues potentially involved in N-linked glycosylation were marked in green. B. The amino acid sequences of predicted serotonin receptors ortologues with best scores in blast searches with the cestode Eca-5-HT1b were aligned using the ClustalW method. The new 5-HT1 type cloned receptors’ names are marked in bold. The transmembrane (TM), intracellular (ICL) and extracellular (ECL) domains are indicated above each alignment. For the sake of simplicity, the amino terminal end, the transmembrane domain 1, the intracellular loop three and the carboxy terminal end were trimmed partially or completely. The position of residues involved in G protein coupling are indicated with asterisks below each alignment. Residues present in the new predicted receptors that were not seen in other GPCRs are underlined. Critical residues involved in ligand binding and receptor function were indicated in bold below each alignment. Cysteine residues potentially involved in disulphide bond formation are marked as S-S between cysteines. The receptor names, identification numbers and the corresponding species from which the receptors were obtained are enlisted in Table C from S1 Text. (TIF) [file pone.0259104.s003.tif]

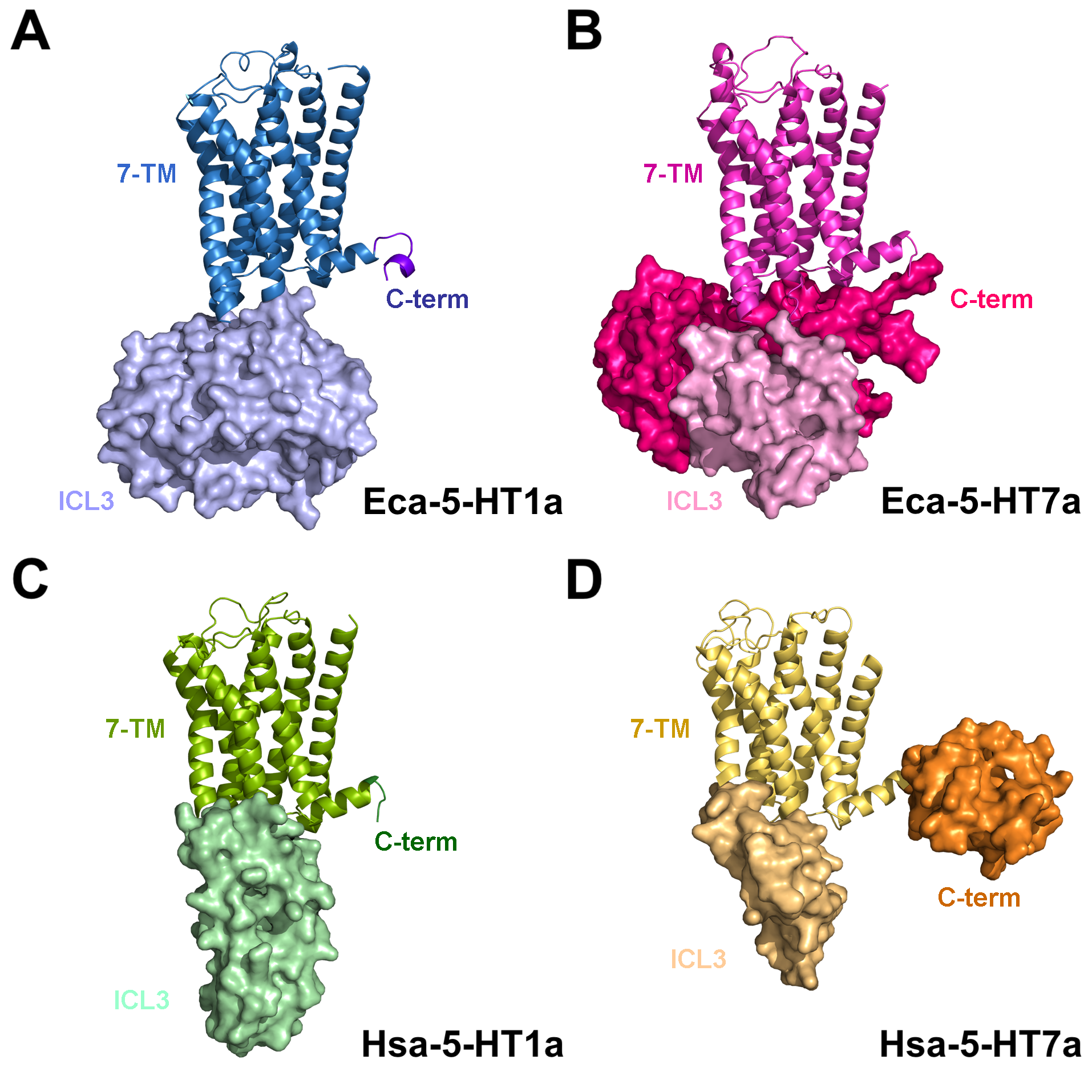

Supplement: S4 Fig — Comparative analysis of the general structures of the homology models to 5-HT1- vs 5-HT7-type serotonergic receptors from Echinococcus canadensis G7 (A) Eca-5-HT1a and (B) Eca-5-HT7a; and Homo sapiens (C) Hsa-5-HT1a and (D) Hsa-5-HT7a. In all the representations, the transmembrane domains I to VII (7-TM) were represented as cartoon while the intracellular loop 3 (ICL3) and C-terminus regions (C-term) were represented as surface. Note the smaller ICL3 and longer carboxy terminal end in 5-HT7-type receptors with respect to 5-HT1-type receptors, in which a bigger ICL3 and smaller C-term (marked as a short cartoon) can be seen. (TIF) [file pone.0259104.s004.tif]

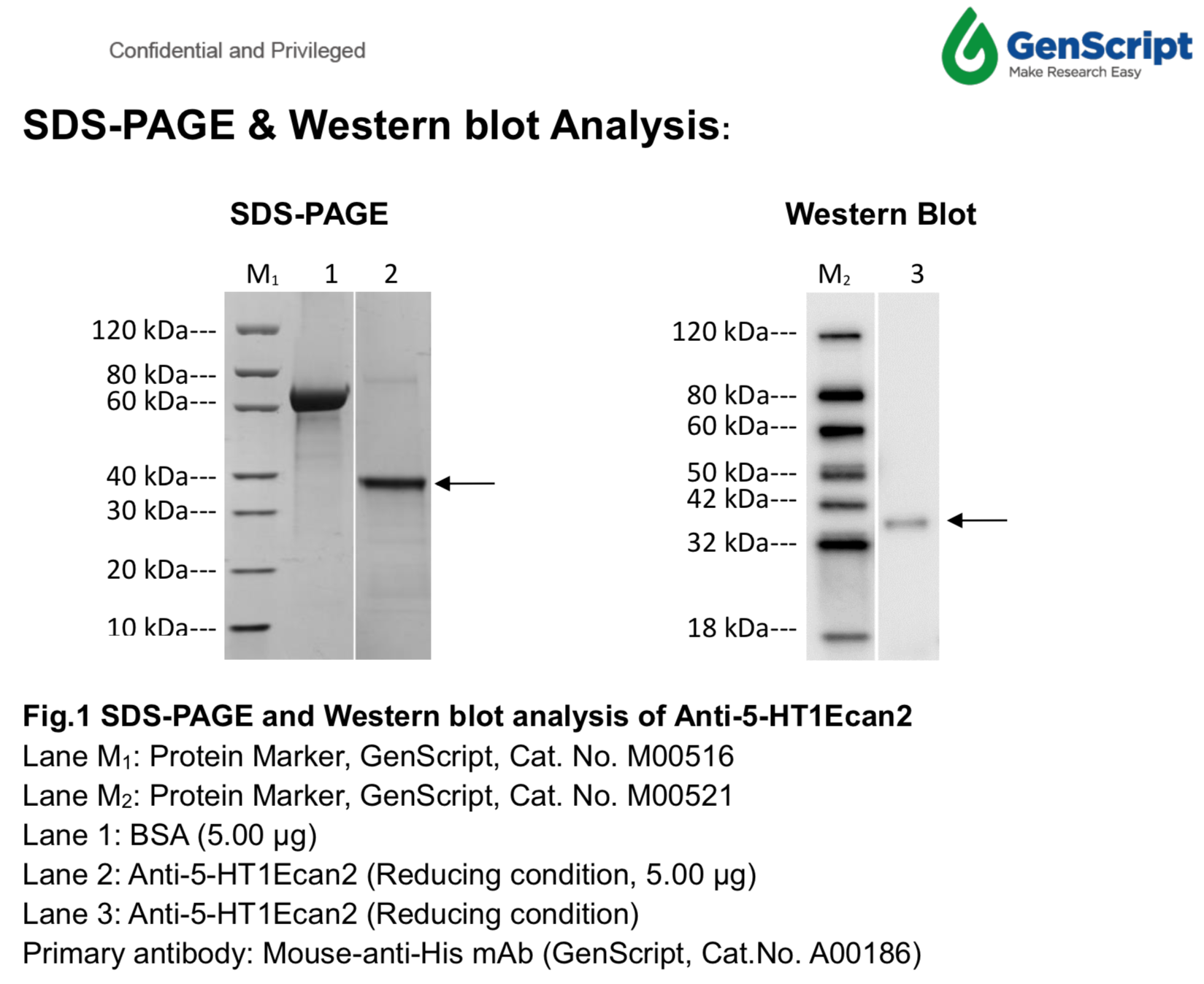

Supplement: S1 Raw image — (TIF) [file pone.0259104.s007.tif]
