# Supplementary material for: Characterization of a new type of neuronal 5-HT G- protein coupled receptor in the cestode nervous system
Source: PLoS One. 2021 Nov 11;16(11):e0259104. doi: 10.1371/journal.pone.0259104 (PMC8584985; doi:10.1371/journal.pone.0259104)
Supplement: S1 Table — (DOCX) [file pone.0259104.s005.docx]

| 5-HT GPCR | G-protein coupling specificity - normalized score (PRED-COUPLE 2.00) | Experimentally validated coupling | Accession number or uniProt ID | Reference^a^ |
| --- | --- | --- | --- | --- |
| Eca-5HT7a | **G_s_ - 0.78** G_i/o_ - **0.68**  G_q/11_ - **0.04** G_12/13_ - **0.04** | G_s_ | MH707372 | [1] |
| Eca-5HT7b | **G_s_ - 0.56**  G_i/o_ - 0.20 G_q/11_ - 0.05 G_12/13_ - 0.00 | G_s_ | MH707373 | [1] |
| Mvo-5HT7a | **Gs - 0.91** G_i/o_ - **0.47**  G_q/11_ - **0.03** G_12/13_ - **0.00** | G_s_ | MH707374 | [1] |
| Sma-5HT7b | **G_s_ - 0.60** G_q/11_ - **0.44**  G_i/o_ - **0.02** G_12/13_ - **0.00** | G_s_ | KX150867 | [2] |
| Cel-5HT1 | **G_i/o_ -** **0.99**  G_s_ - **0.01** G_q/11_ - **0.00** G_12/13_ - **0.00** | G_i/o_ | G5EGH0 | [3] |
| Cel-5HT2 | **G_q/11_ -** **0.90**  G_s_ - **0.22** G_i/o_ - **0.07** G_12/13_ - **0.00** | G_q/11_ | O17470 | [4] |
| Cel-5HT7 | G_s_ - **0.94**  G_q/11_ - **0.11** G_i/o_ - **0.09** G_12/13_ - **0.01** | G_s_ | Q22895 | [5] |
| Dme-5HT1a | G_i/o_ - **0.99** G_s_ - **0.00** G_q/11_ - **0.00** G_12/13_ - **0.00** | G_i/o_ | CAA77570.1 | [6] |
| Dme-5HT1b | G_i/o_ - **0.83** G_q/11_ - **0.12** G_s_ - **0.01** G_12/13_ - **0.00** | G_i/o_ | CAA77571.1 | [6] |
| Dme-5HT7 | G_s_ - **0.77** G_i/o_ - **0.42** G_12/13_ - **0.05** G_q/11_ - **0.02** | G_s_ | NP_524599.1 | [7] |

^a^Full references

[1] Camicia F, Celentano AM, Johns ME, Chan JD, Maldonado L, Vaca H, Di Siervi N, Kamentezky L, Gamo AM, Ortega-Gutierrez S, Martin-Fontecha M, Davio C, Marchant JS, Rosenzvit MC. Unique pharmacological properties of serotoninergic G-protein coupled receptors from cestodes. PLoS Negl Trop Dis. 2018 Feb 9;12(2):e0006267. doi: 10.1371/journal.pntd.0006267

[2] Chan JD, McCorvy JD, Acharya S, Johns ME, Day TA, Roth BL, Marchant JS. A Miniaturized Screen of a *Schistosoma mans*oni Serotonergic G Protein-Coupled Receptor Identifies Novel Classes of Parasite-Selective Inhibitors. PLoS Pathog. 2016 May 17;12(5):e1005651. doi: 10.1371/journal.ppat.1005651

[3] Olde B, McCombie WR. Molecular cloning and functional expression of a serotonin receptor from *Caenorhabditis elegans*. J Mol Neurosci. 1997 Feb;8(1):53-62. doi: 10.1007/BF02736863

[4] Hamdan FF, Ungrin MD, Abramovitz M, Ribeiro P. Characterization of a novel serotonin receptor from *Caenorhabditis elegans*: cloning and expression of two splice variants. J Neurochem. 1999 Apr;72(4):1372-83. doi: 10.1046/j.1471-4159.1999.721372.x

[5] Hobson RJ, Hapiak VM, Xiao H, Buehrer KL, Komuniecki PR, Komuniecki RW. SER-7, a *Caenorhabditis elegans* 5-HT7-like receptor, is essential for the 5-HT stimulation of pharyngeal pumping and egg laying. Genetics. 2006 Jan;172(1):159-69. doi: 10.1534/genetics.105.044495

[6] Saudou F, Boschert U, Amlaiky N, Plassat JL, Hen R. A family of *Drosophila* serotonin receptors with distinct intracellular signalling properties and expression patterns. EMBO J. 1992 Jan;11(1):7-17

[7] Witz P, Amlaiky N, Plassat JL, Maroteaux L, Borrelli E, Hen R. Cloning and characterization of a *Drosophila* serotonin receptor that activates adenylate cyclase. Proc Natl Acad Sci U S A. 1990 Nov;87(22):8940-4. doi: 10.1073/pnas.87.22.8940
