# Supplementary material for: Characterization of a new type of neuronal 5-HT G- protein coupled receptor in the cestode nervous system
Source: PLoS One. 2021 Nov 11;16(11):e0259104. doi: 10.1371/journal.pone.0259104 (PMC8584985; doi:10.1371/journal.pone.0259104)
Supplement: S1 Text — (DOCX) [file pone.0259104.s008.docx]

**Original sequence**

>eca-5-HT_1a_icl3

TGCTCGTCGTCATATTAGGAAGAAACATTTTGTCAAATACCAAACCAATTCGACCACCATAAAGGGCAACAGTTATCAGTCGGCTACCTCTGCTGAAAATCTGGAAAGCCACCGTTATTGCTGTTACTGCTGTGGCTACGTCATGGAGACAAGATTTCACAAGGGCAGGACAATGTTCAACGTGGATTCTACCCAGTGCTCAGGATTCCAATGTTCTAGCTCCTCCGATAAAAGGATGGTAGCCACGGACCCCCCTGAAACCGTTGTTGCAGCAGCGGTGACGGCCATGCGTATAGCTGCGACCATGATTAGCACCACAAAGGAACCCGAGTGGGGGACCTACGAATCCGAAAGCTCAGACAGTGAAGGGACATTTAATTTGATCTCCTTTAACTCTTTAAAGAATCCAAATTATGATAATGAGTCCCAATTCTATGCAAGTAGGCAAATGAATTGCCAGCCCTTGACGTATGATACTCTGCGCAGTCTGCGGAAGCAAAAAGAGCTGGGTTCTAACCGAGAAGTAATAGCATCTCCATCGGGTAAAGAACTGGTGCGTGAGCTTTCTAGCTACACAAATTGTTCCTCCATTCTGTATGCAGATGAAAATTCCAAACCCAATTCCCTTTCCTCTAGTGTGTACTCCTCAATGATACACACCTCCGACTCGAGGGAGGTAGAGAACGGAAACACTACTGAAGAGCCTCAACTACGCAACTTTGTTCCTCTGTGCATTTACTACAAAAAACAGAGACAACAACGTTGGTTAGCACAAGGAAGGATTTTTAATAAGAAGGCTAGTGAAGTTATGAAGAATTTGTCAGTAAACGAGTCAGATTCAGGTATAAAGCCCGAGGAGGAGCGCTACCTGCGAGAGCGTCTAGAGCAAAGGCGCGAACGAAAAACG

**Nucleotide optimized sequence**

>eca-5-HT_1a_icl3opt

ACAGCAAGGAGACACATAAGGAAAAAACACTTCGTGAAATACCAAACCAATAGCACCACCATCAAAGGTAACAGCTACCAGAGCGCGACCAGCGCGGAGAACCTGGAAAGCCACCGTTACTGCTGCTATTGCTGCGGTTATGTGATGGAAACCCGTTTCCACAAGGGCCGTACCATGTTTAACGTTGATAGCACCCAGTGCAGCGGCTTCCAATGCAGCAGCAGCAGCGACAAACGTATGGTGGCGACCGATCCGCCGGAAACCGTGGTTGCGGCGGCGGTTACCGCGATGCGTATTGCGGCGACCATGATTAGCACCACCAAGGAGCCGGAATGGGGTACCTACGAGAGCGAAAGCAGCGACAGCGAGGGCACCTTCAACCTGATTAGCTTTAACAGCCTGAAAAACCCGAACTACGATAACGAAAGCCAGTTTTATGCGAGCCGTCAGATGAACTGCCAACCGCTGACCTATGACACCCTGCGTAGCCTGCGTAAGCAAAAAGAGCTGGGTAGCAACCGTGAAGTGATTGCGAGCCCGAGCGGCAAGGAGCTGGTTCGTGAACTGAGCAGCTACACCAACTGCAGCAGCATTCTGTATGCGGATGAGAACAGCAAACCGAACAGCCTGAGCAGCAGCGTGTACAGCAGCATGATCCACACCAGCGACAGCCGTGAGGTTGAAAACGGTAACACCACCGAGGAACCGCAACTGCGTAACTTCGTGCCGCTGTGCATCTACTATAAGAAACAGCGTCAGCAACGTTGGCTGGCGCAAGGTCGTATTTTTAACAAGAAAGCGAGCGAGGTGATGAAGAACCTGAGCGTTAACGAAAGCGACAGCGGCATCAAACCGGAAGAAGAGCGTTATCTGCGTGAGCGTCTGGAACAGCGTCGTGAGCGTAAAACC

**Amino acidic deduced sequence**

>Eca-5-HT_1a_ICL3

TARRHIRKKHFVKYQTNSTTIKGNSYQSATSAENLESHRYCCYCCGYVMETRFHKGRTMFNVDSTQCSGFQCSSSSDKRMVATDPPETVVAAAVTAMRIAATMISTTKEPEWGTYESESSDSEGTFNLISFNSLKNPNYDNESQFYASRQMNCQPLTYDTLRSLRKQKELGSNREVIASPSGKELVRELSSYTNCSSILYADENSKPNSLSSSVYSSMIHTSDSREVENGNTTEEPQLRNFVPLCIYYKKQRQQRWLAQGRIFNKKASEVMKNLSVNESDSGIKPEEERYLRERLEQRRERKT
