# Supplementary material for: Characterization of a new type of neuronal 5-HT G- protein coupled receptor in the cestode nervous system
Source: PLoS One. 2021 Nov 11;16(11):e0259104. doi: 10.1371/journal.pone.0259104 (PMC8584985; doi:10.1371/journal.pone.0259104)
Supplement: S2 Text — (DOCX) [file pone.0259104.s009.docx]

**Table 1. Parameters determined for the structure homology models generated in this work.**

| **Names** | **ERRAT** | **QMEAN** | **RMSD^a^** | **TM-align^a^** | **Ramachandram Plot** | |
| --- | --- | --- | --- | --- | --- | --- |
|  |  |  |  |  | **Favoured** | **Outliers** |
| Hsa-5-HT1b | 100 | -3.62 | - | - | 97.4% | 0.0% |
| Eca-5-HT1a | 95.3805 | -4.21 | 0.65 | 0.93049 | 95.7% | 0.4% |
| Mvo-5-HT1a | 92.9263 | -4.24 | 0.65 | 0.93084 | 96.0% | 0.7% |
| Hmi-5-HT1a | 96.5318 | -3.43 | 0.73 | 0.92725 | 96.7% | 0.4% |
| Eca-5-HT7a | 96.1451 | -3.79 | 0.57 | 0.93846 | 95.2% | 1.1% |
| Mvo-5-HT7a | 95.3718 | -4.00 | 0.27 | 0.93977 | 95.2% | 1.1% |
| Hmi-5-HT7a | 96.1749 | -3.93 | 0.24 | 0.93758 | 96.0% | 0.7% |
| Hsa-5-HT1a | 92.2764 | -4.13 | 0.15 | 0.95292 | 96.5% | 0.0% |
| Hsa-5-HT7a | 82.9876 | -4.97 | 1.8 | 0.87781 | 91.8% | 1.6% |
| ^a^ These parameters were determined by comparing the seven-transmembrane domain of the structure homology models performed here with Hsa-5-HT1b (PDB: 4IAR) | | | | | | |

**
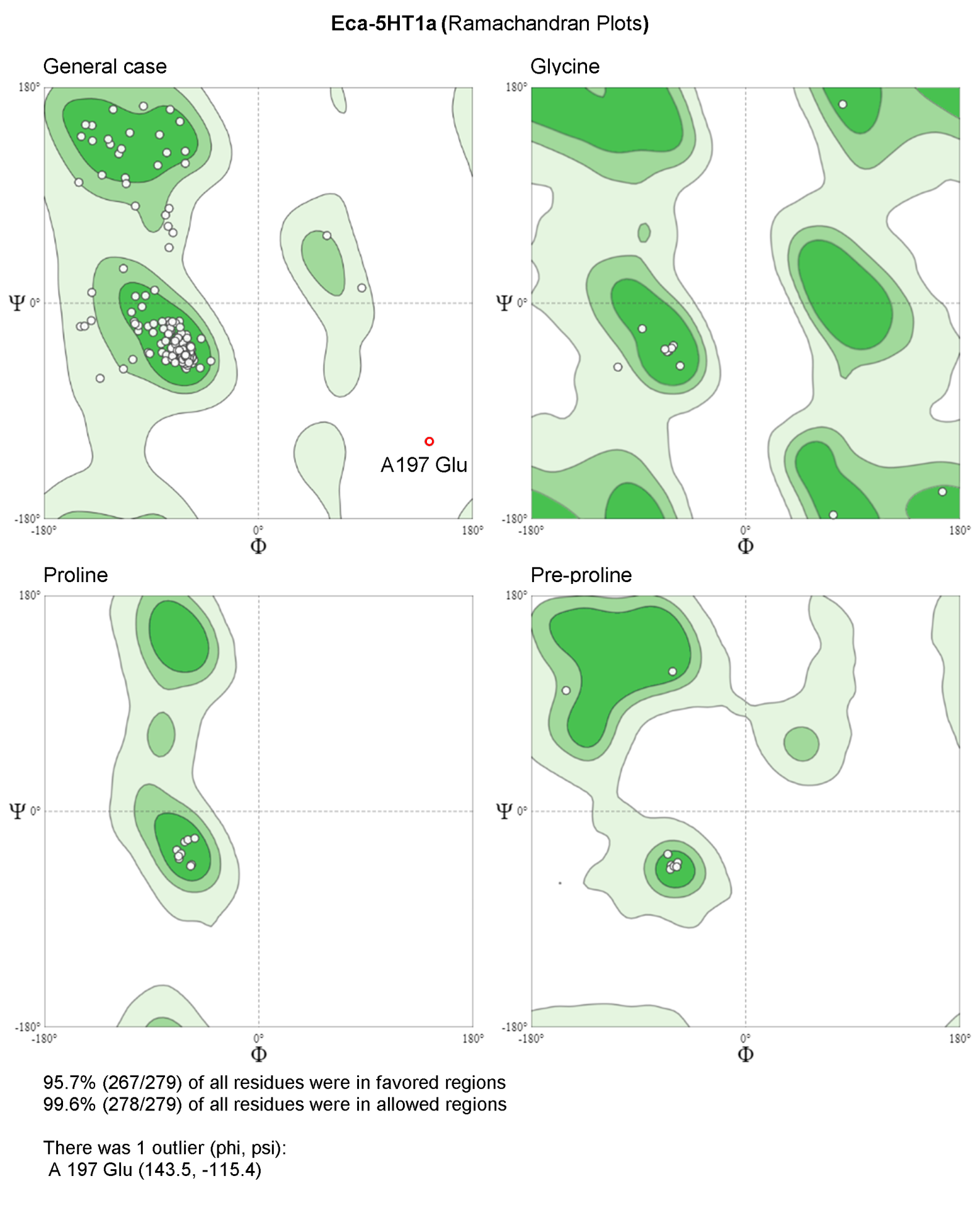

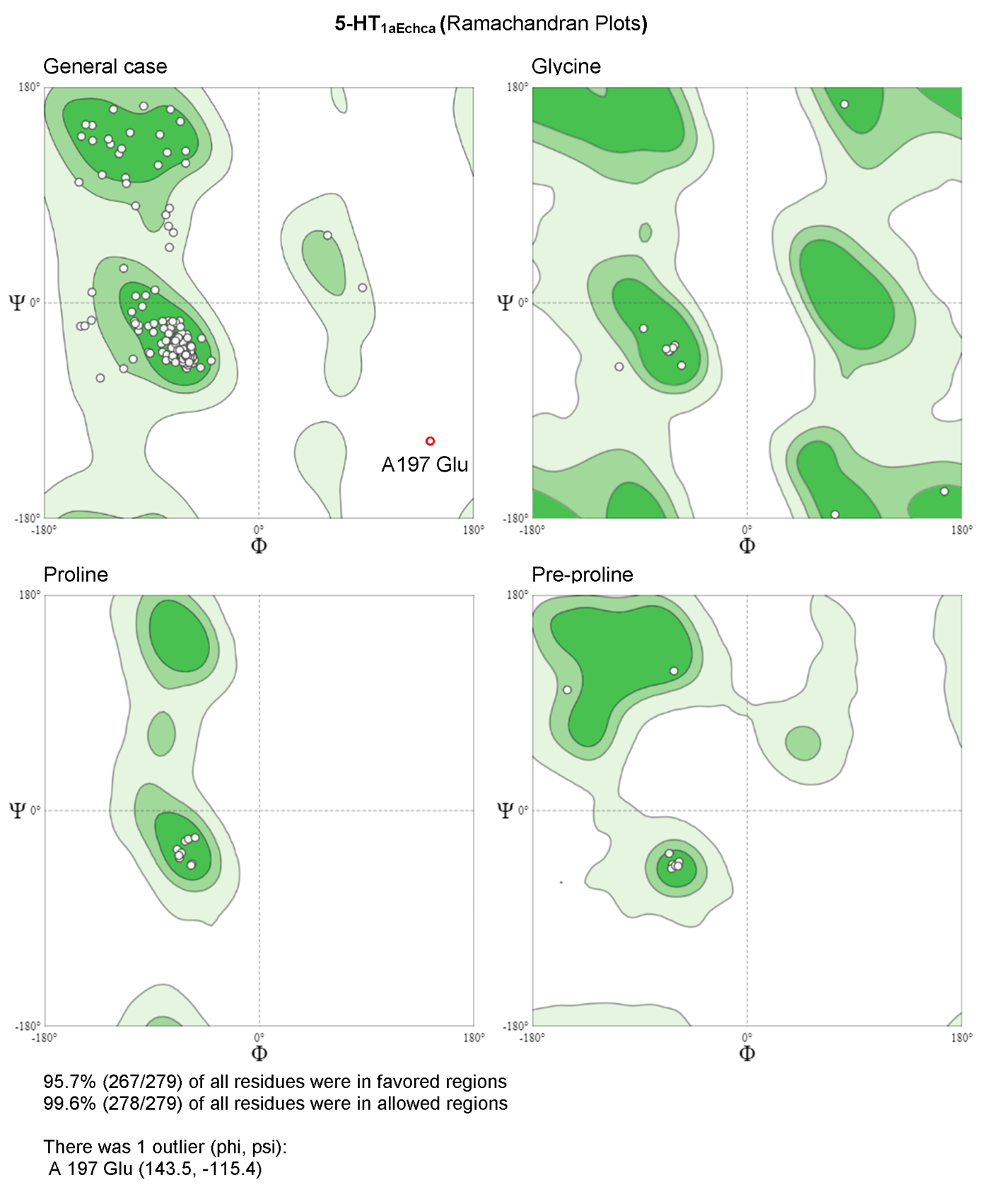
Figure A. Ramachandran plots calculated for the homology model of Eca-5-HT1a receptor.**

**Eca-5-HT1a**

**Figure B. Ramachandran plots calculated for the homology model of Mvo-5-HT1a receptor.**

**Mvo-5-HT1a**

**
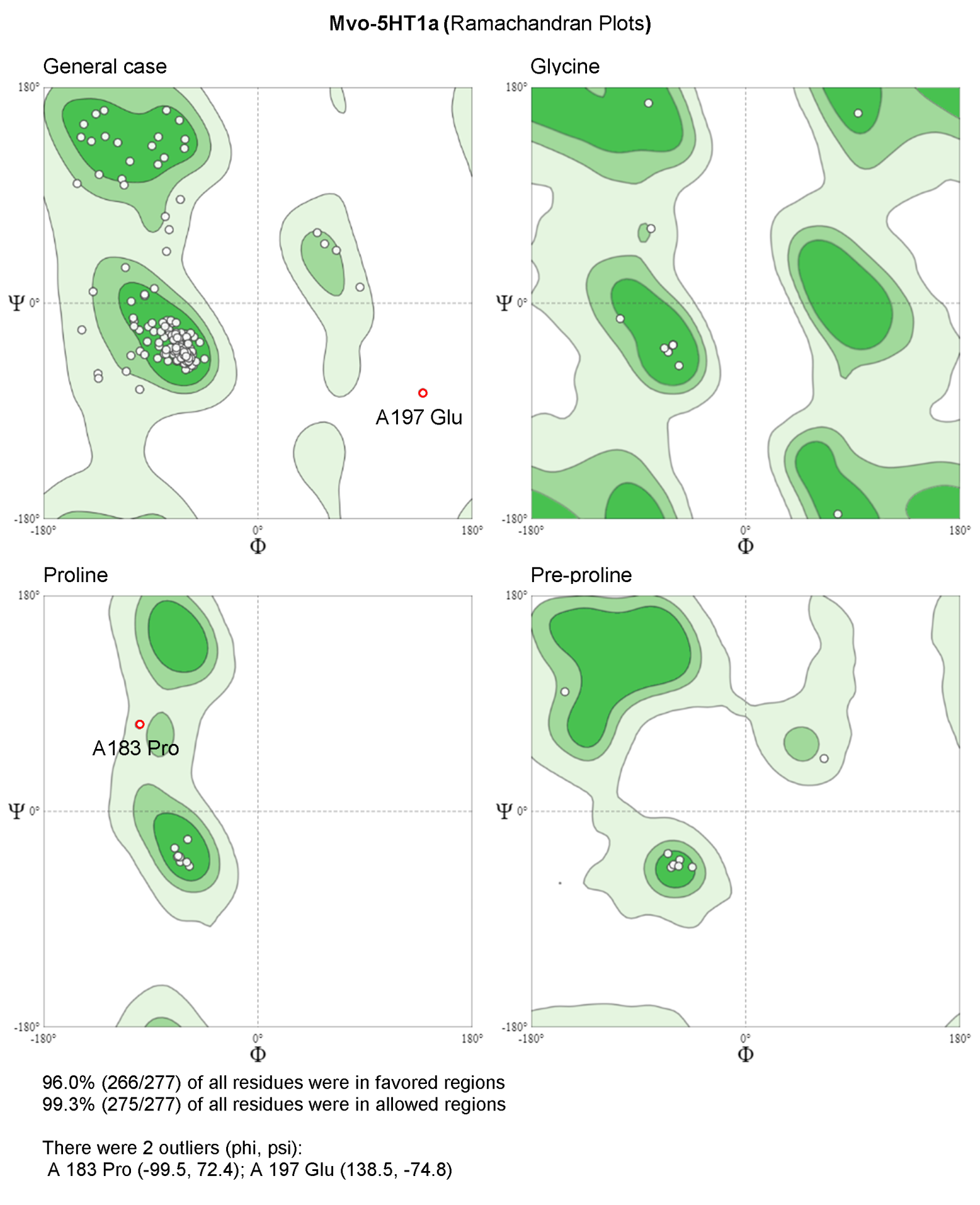

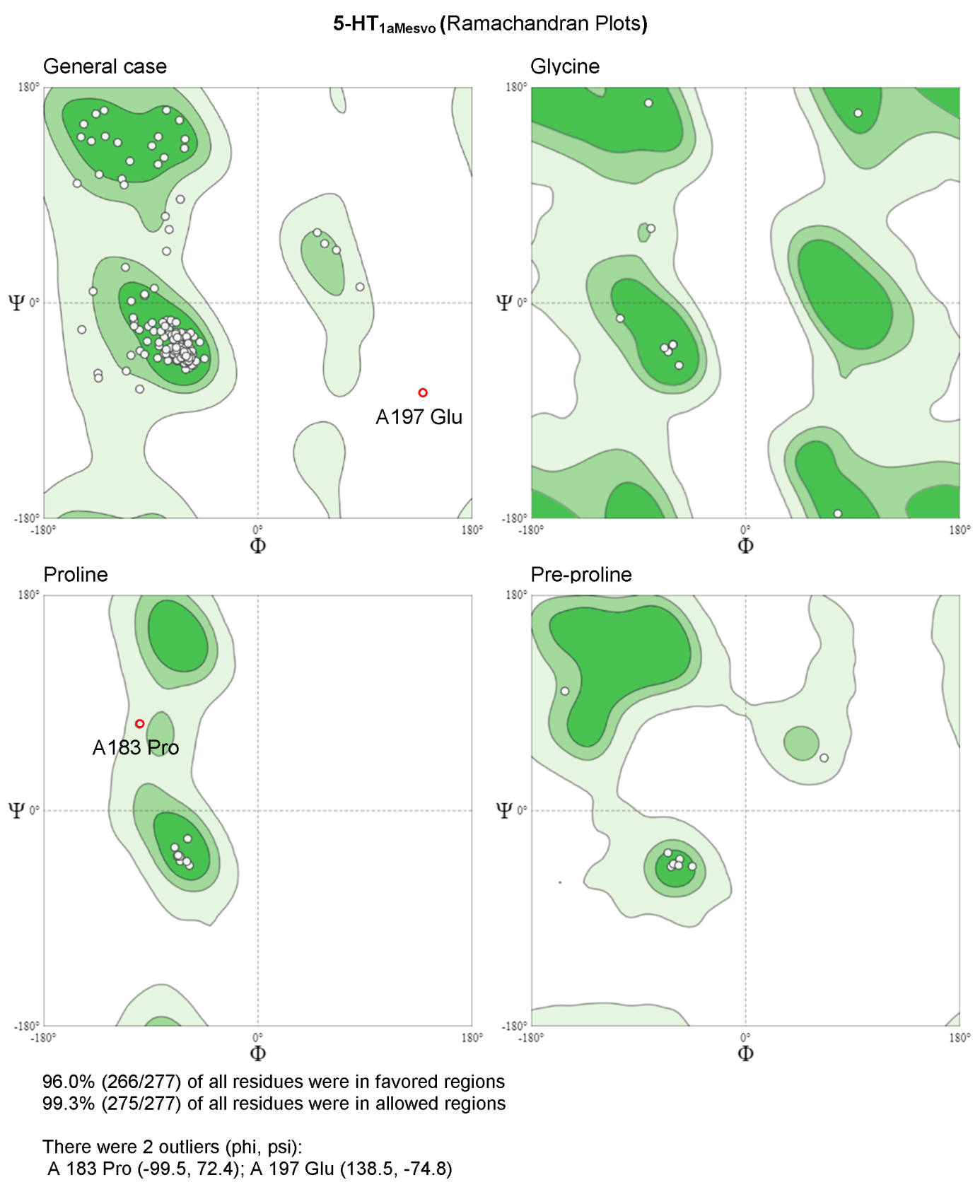
**

**Figure C. Ramachandran plots calculated for the homology model of Hmi-5-HT1a receptor.**

**Hmi-5-HT1a**

**
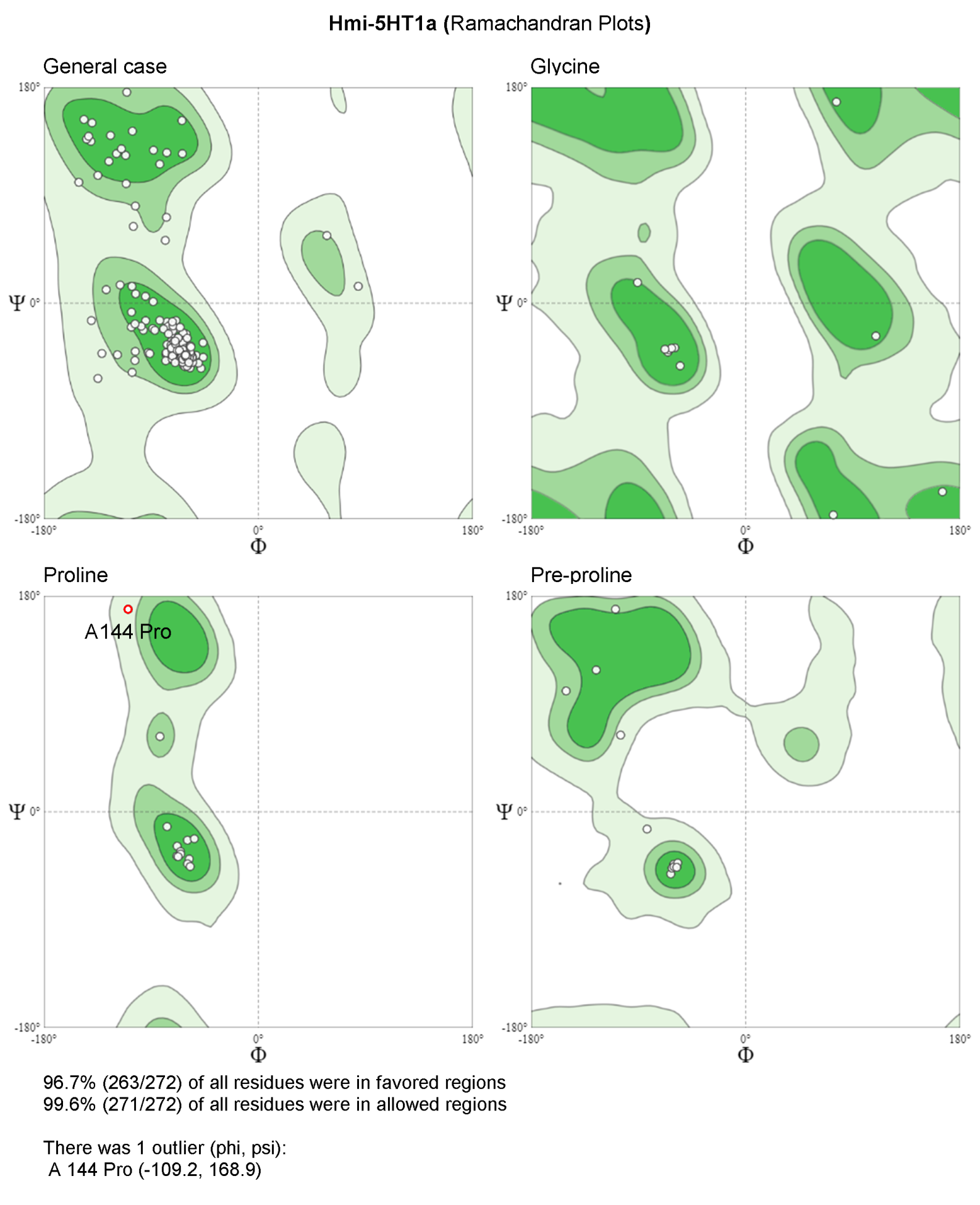

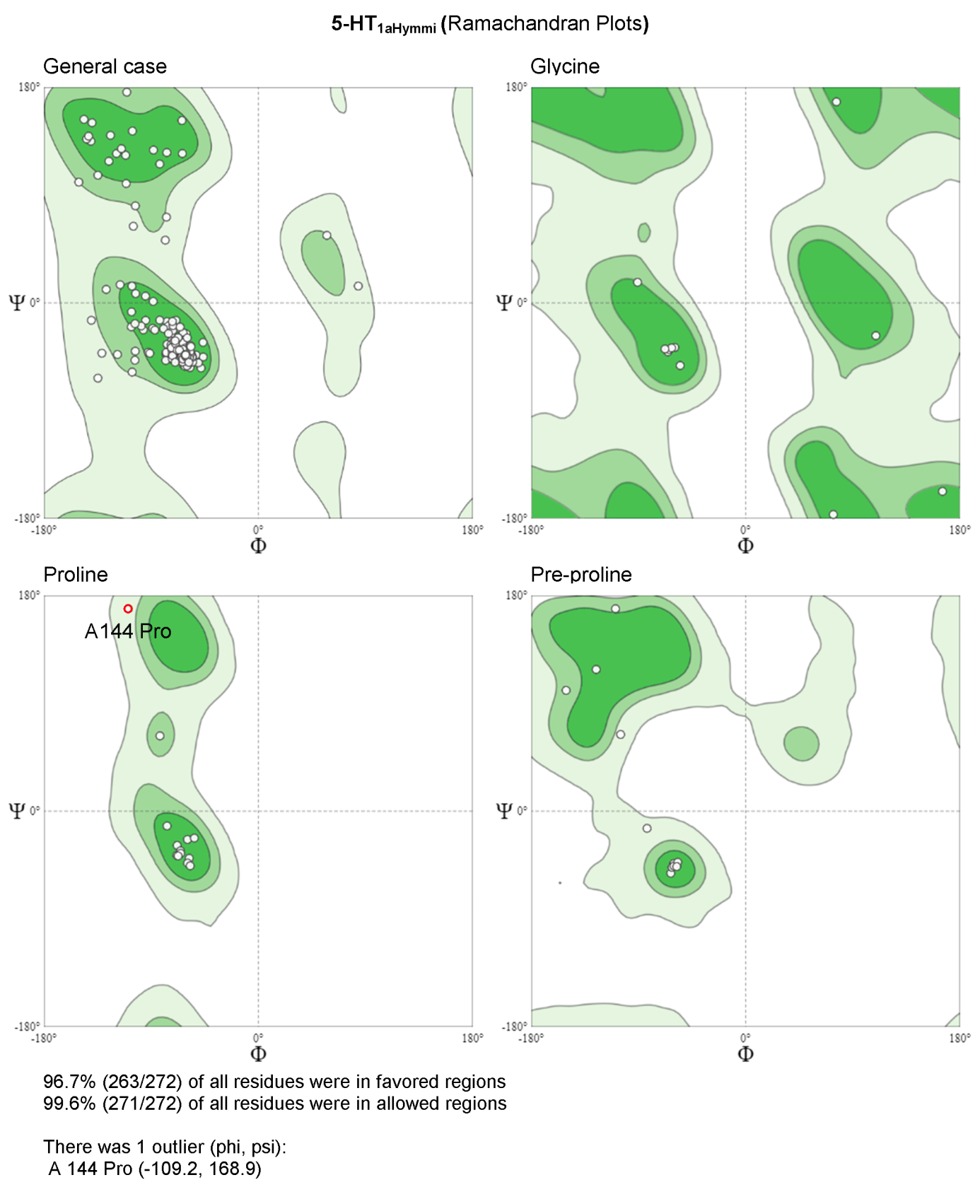
**

**Figure D. Ramachandran plots calculated for the homology model of Eca-5-HT7a receptor.**

**Eca-5-HT7a**

**
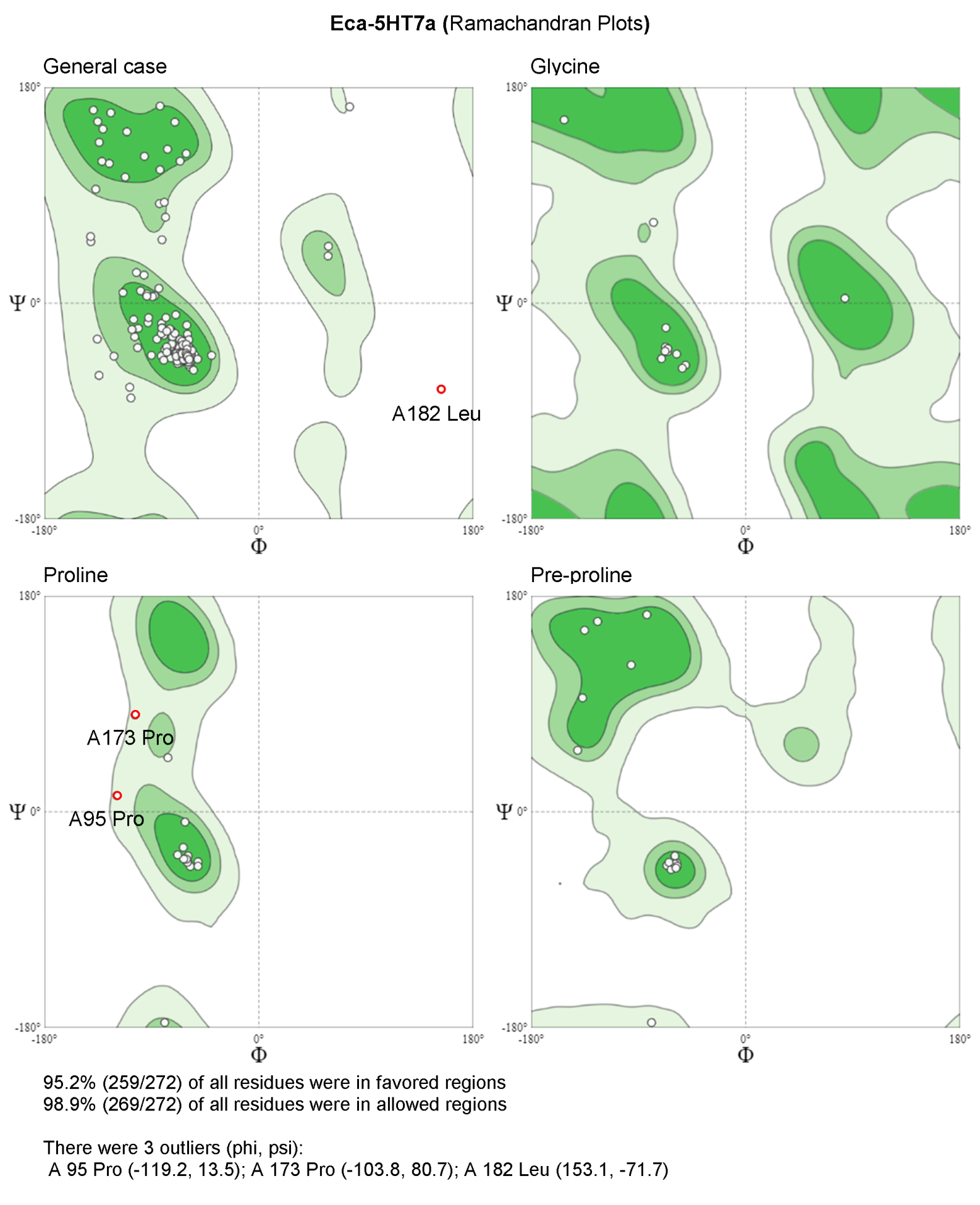

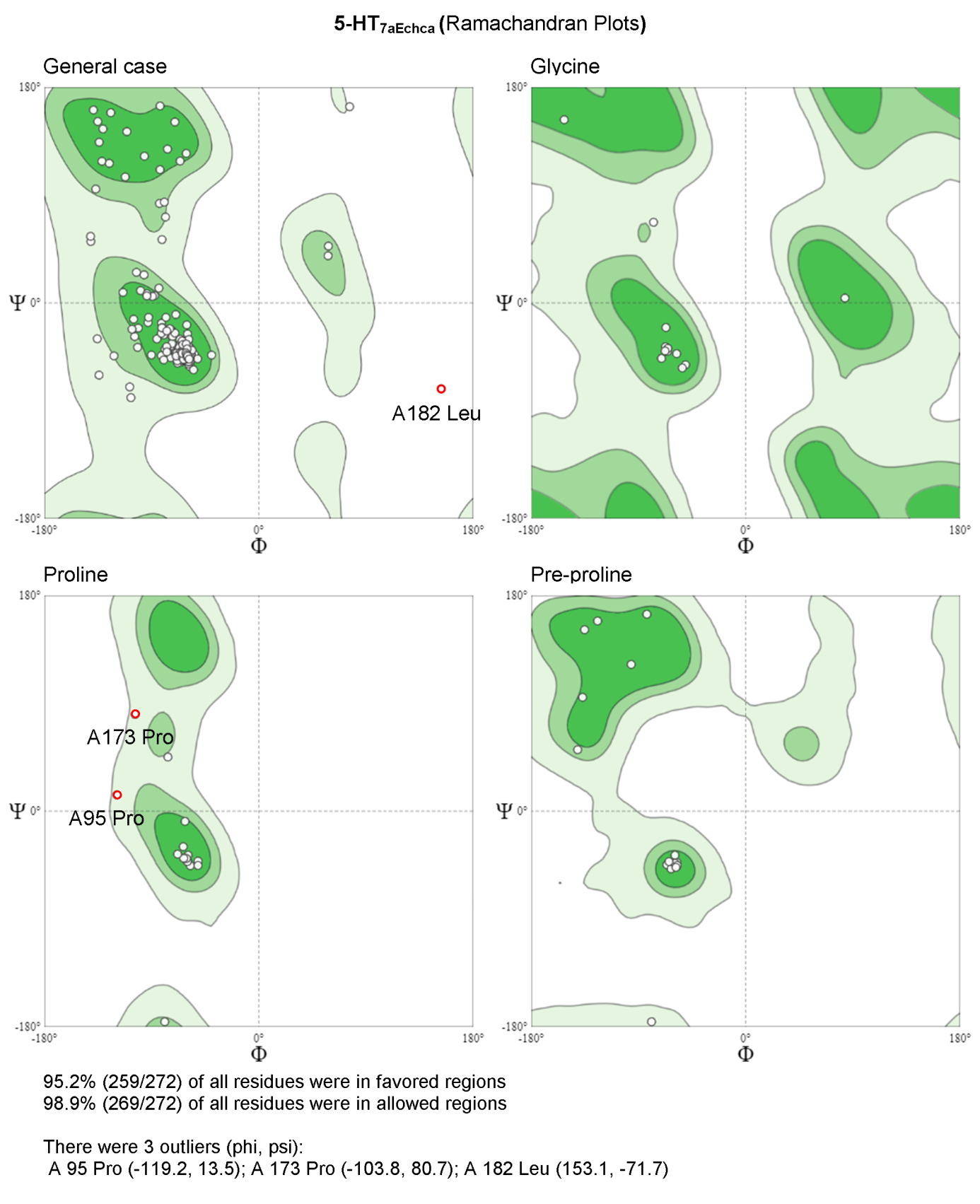
**

**Figure E. Ramachandran plots calculated for the homology model of Mvo-5-HT7a receptor.**

**Mvo-5-HT7a**

**
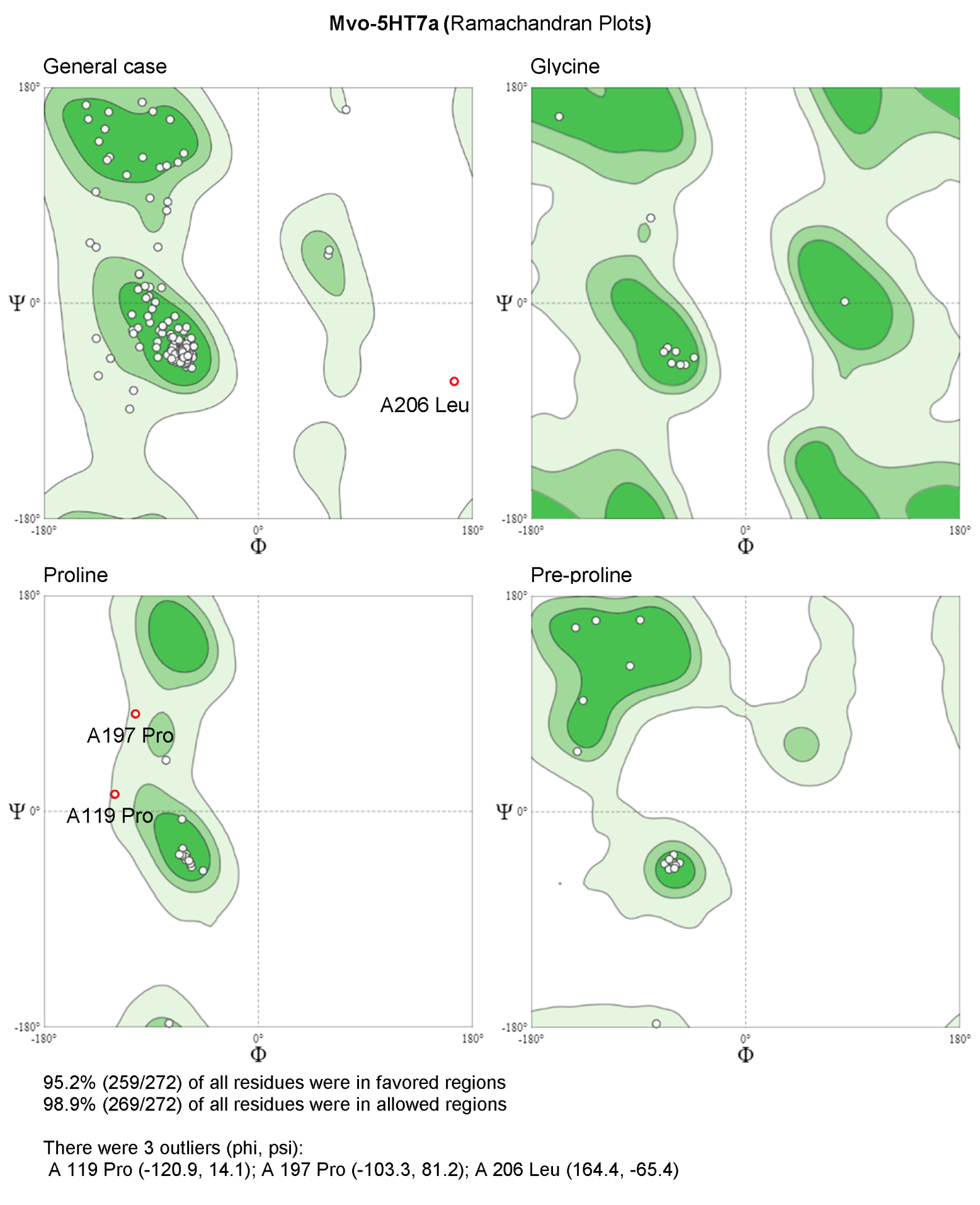

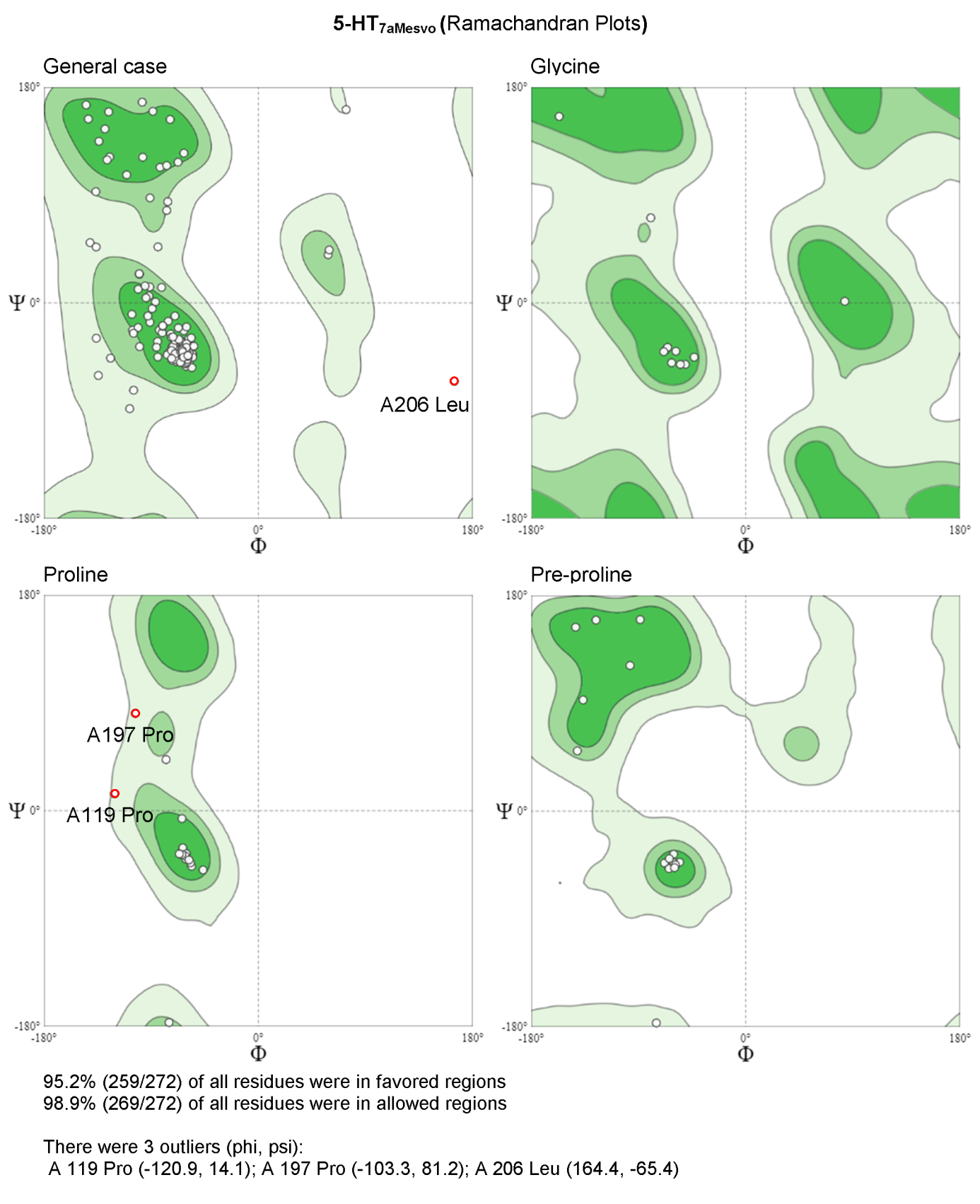
**

**Figure F. Ramachandran plots calculated for the homology model of Hmi-5-HT7a receptor.**

**Hmi-5-HT7a**

**
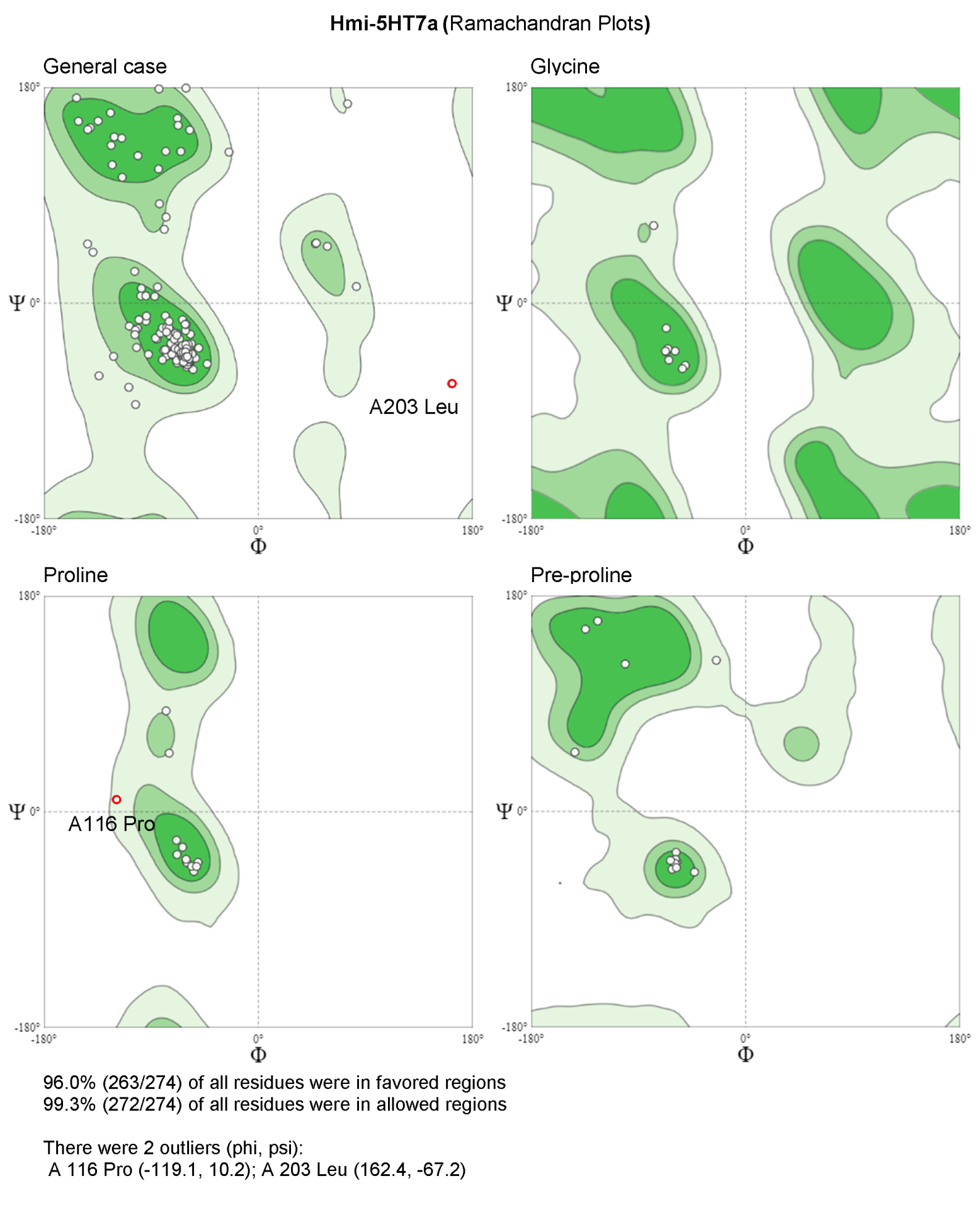

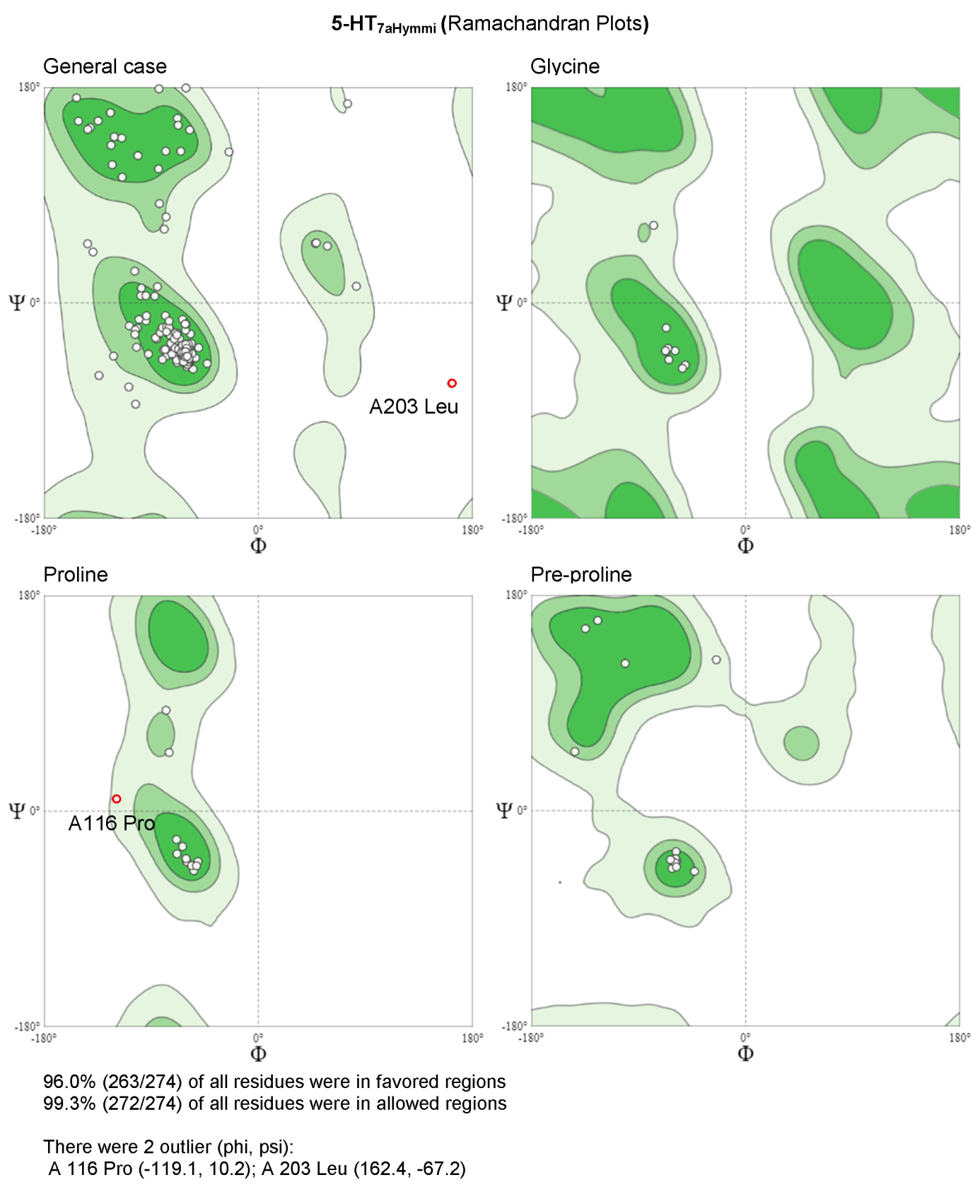
**

**Figure G. Ramachandran plots calculated for the homology model of Hsa-5-HT1a receptor.**

**Hsa-5-HT1a**

**
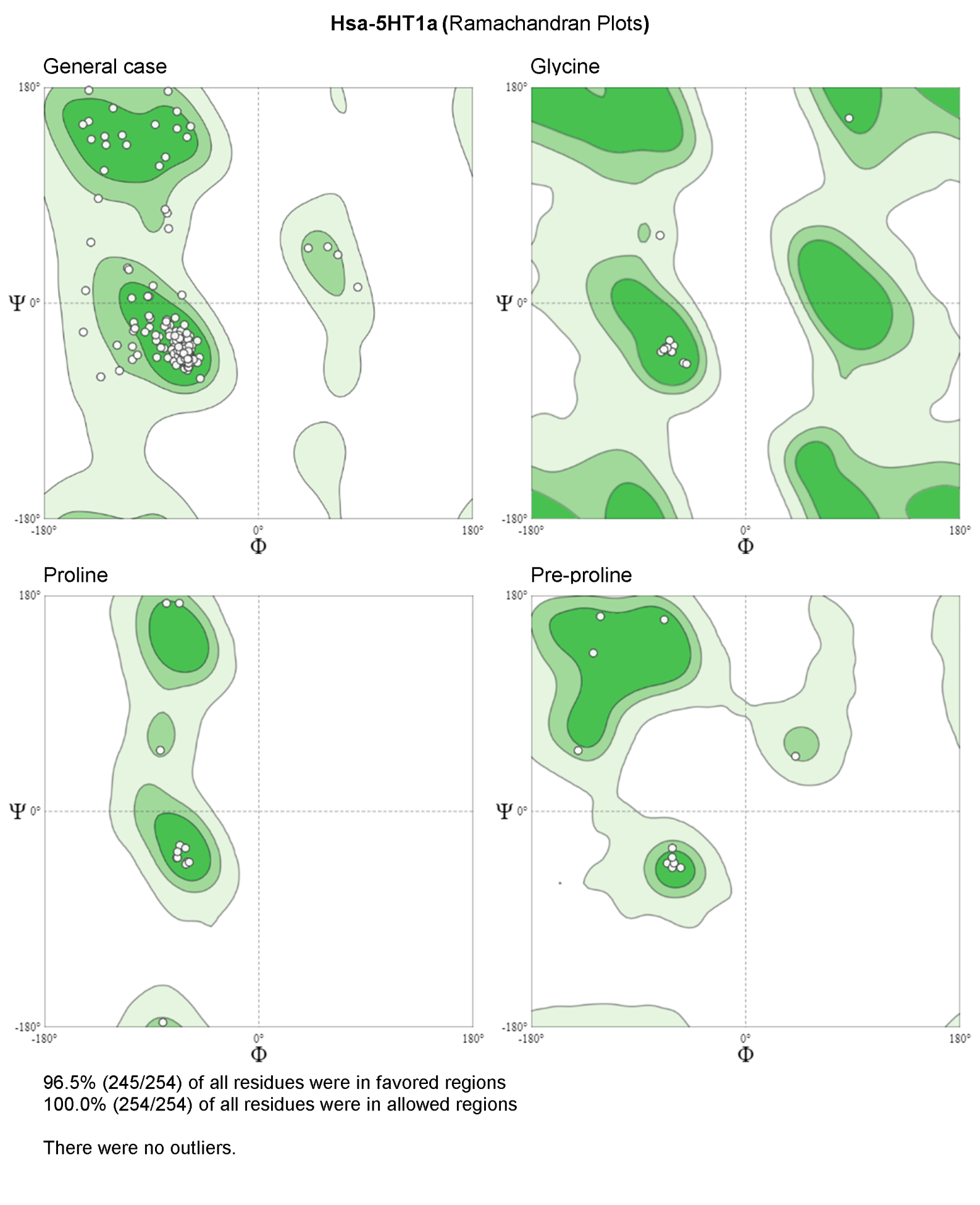
**

**Figure H. Ramachandran plots calculated for the homology model of Hsa-5-HT7a receptor.**

**Hsa-5-HT7a**

**
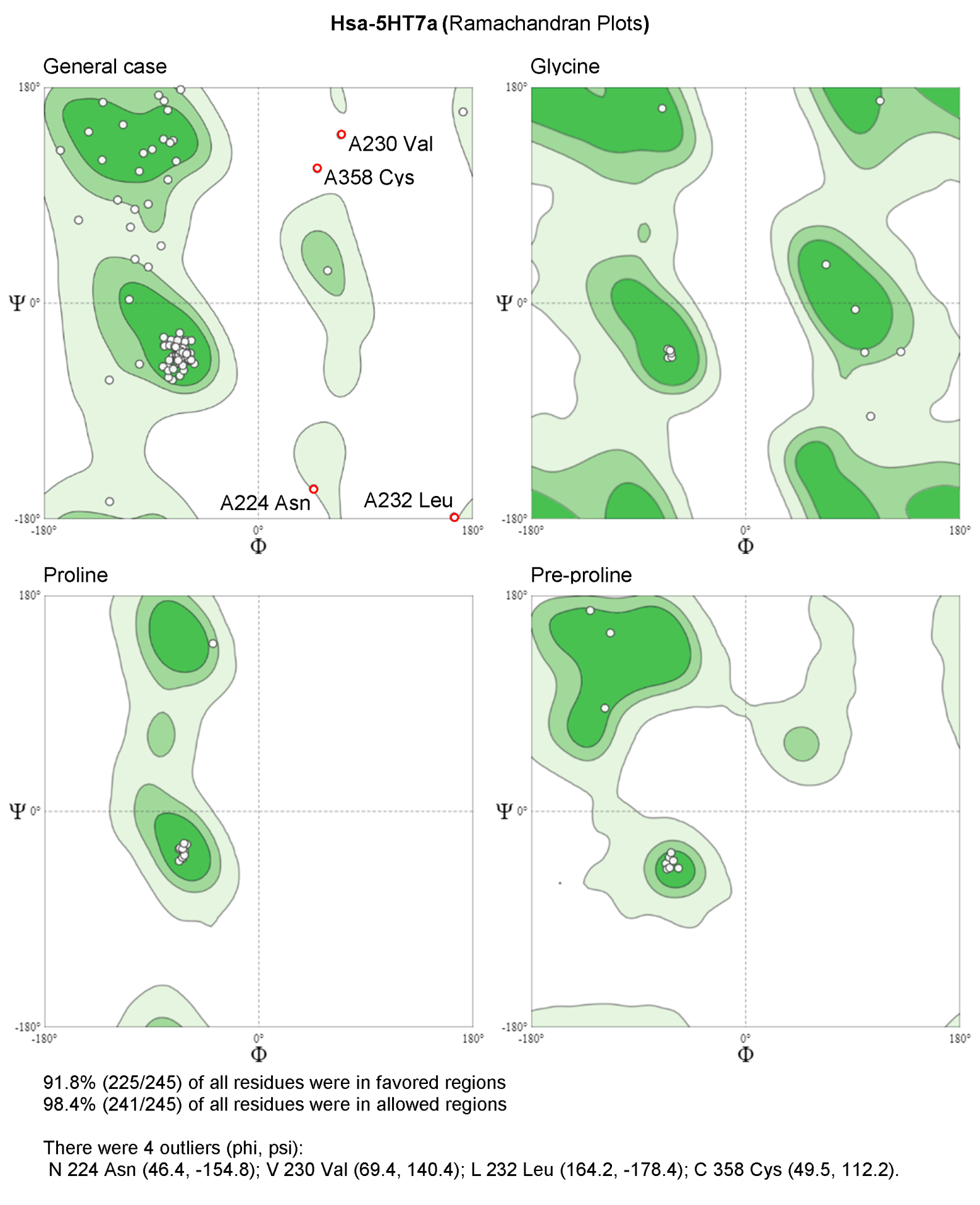
**
