# Supplementary material for: Characterization of a new type of neuronal 5-HT G- protein coupled receptor in the cestode nervous system
Source: PLoS One. 2021 Nov 11;16(11):e0259104. doi: 10.1371/journal.pone.0259104 (PMC8584985; doi:10.1371/journal.pone.0259104)
Supplement: Attachment — Submitted filename: Response to reviewers 2021 3 31.docx [file pone.0259104.s010.docx]

April 12, 2021

**Dear Editor and Reviewers,**

We are grateful for all your constructive input into our manuscript and will, in the following pages, provide answers to your questions and concerns. A revised manuscript, including tracked changes, as well as a final version, are attached to the submission.

Sincerely yours,

**Dr Mara C. Rosenzvit**

IMPAM-UBA-CONICET, Facultad de Medicina - Universidad de Buenos Aires Paraguay 2155, piso 13 (1121) Buenos Aires – ARGENTINA
Tel: (5411) 5950-9500 ext. 2192 Fax: (5411) 5950-9577
E-mail: mrosenzvit@fmed.uba.ar; mararosenzvit@gmail.com

**Professor Dr. Jonathan S. Marchant**

Marcus Professor & Chair
Department of Cell Biology, Neurobiology & Anatomy 8701 Watertown Plank Road
Post Office Box 26509
Milwaukee, Wisconsin 53226-0509
Tel: (414) 955-8261
Fax: (414) 955-6517
jmarchant@mcw.edu

**Responses to the reviewers' comments**

Reviewer's Responses to Questions

**Key Review Criteria Required for Acceptance?**

As you describe the new analyses required for acceptance, please consider the following:

**Methods**

-Are the objectives of the study clearly articulated with a clear testable hypothesis stated?

-Is the study design appropriate to address the stated objectives?

-Is the population clearly described and appropriate for the hypothesis being tested?

-Is the sample size sufficient to ensure adequate power to address the hypothesis being tested?

-Were correct statistical analysis used to support conclusions?
-Are there concerns about ethical or regulatory requirements being met?

**Reviewer #1:** Appropriate methods used, very detailed description of methodology provided. Work could be readily repeated from the description.

**Reviewer #2:** The objectives of the study are clar, the studies are well designed. There are a few questions to the authors (see below).

**Reviewer #3:** The objectives of the study are clear and the methods used are adequate to address claim. These are methods that the authors and others in the field have used to publish similar findings. The authors claim to have identified and functionally characterised a novel 5HT receptor belonging to type 1 group of 5HT receptors which they have named 5-HT1aEchca.  The *in silico* methods used to characterise 5-HT1aEchca are extremely detailed and produce convincing evidence that this receptor is in fact type 1. The authors also identify a second novel 5HT receptor, 5-HT1bEchca, which is not functionally characterised in this study due to expression issues, however, authors present very little detail of the bioinformatic analysis carried out for this receptor. Further *in silico* analysis of this receptor is warranted to state that it also belongs to type 1 grouping as outlined in the manuscript. The authors include motifs for this additional receptor in results tables but do not show evidence of other bioinformatic analyses such as alignments, homology or structure or describe it in comparison to 5-HT1aEchca, however, still state that this is also a type 1 receptor and previously uncharacterised. A similar level of detailed analysis is required for this receptor is needed if it is to be included in the manuscript results.   
Thank you very much for this interesting suggestion. We have analyzed the Eca-5HT1b receptor further as suggested. In this new version we have provided a more detailed description of this receptor. The phylogeny obtained (Fig 1, Panel C), the multiple sequence alignments with other 5HT1 receptors (S2 Fig), the residues observed at the ligand binding sites (S2 Fig, panel B and Table 1) and coupling regions (S2 Fig, panel B and Table 2), the general structure of this hypothetical receptor (with biggest intracellular loop and smaller carboxy terminal end than their 5HT7 counterparts, Table 3 and S4 Fig) and finally, the high score obtained with the PRED COUPLE 2.00 program are provided (Table 4). Other analyses included the gene structure, with the exon and introns numbers, the hypothetical size of the messenger RNA, protein length and molecular weight of the protein predicted. All these results were now included in the revised version of the manuscript. See lines 613 to 835 and also 1093 to 1096 in the “Revised Manuscript with Changes Highlighted” version.

*In silico* analysis of 5-HT1aEchca does show evidence that this receptor is type 1, however, efforts to functionally characterise as a type 1 receptor were unsuccessful. Authors outline that additional experimental investigations are required. In the discussion of the manuscript an agonist for type 1 5HT receptors is outlined (UCM2550) as having an impact on protocoleces therefore suggesting it binds to Cestode type 1 5HT receptors. An additional and easy experiment to functionally prove that this receptor is type 1 would be to include this agonist (or other agonists/antagonists from literature) as part of the heterologous expression experiments. Pre-treating or creating competition with the 5HT using UCM2550 or other compounds should help validate hypotheses that this receptor is specific for serotonin and type 1. Without functional characterisation as type 1 authors should change conclusions to reflect these outcomes, stating hypothesized type 1 grouping based on bioinformatic analysis. Currently functional data simply proves the receptor is activated by 5HT.
Thank you for this interesting suggestion. All our attempts to pharmacological profile 5-HT1aEchca (or Eca-5HT1a in this new version) were unsuccessful. Agonists like 8OH-DPAT, sumatriptan, quipazine were already tested but without activity:

**Figure: Dose response profile of Eca-5HT1a in the HEK293-GNA15 stable cell line.**

Left panel. Time resolved measurement of Ca^2+^ accumulation in HEK293-GNA15 cells incubated with the indicated concentrations of serotonin, A; or serotonergic agonists, Sumatriptan, B; 8OH-DPAT, C and Quipazine, D. Right panel. Time resolved measurement of Ca^2+^ accumulation in Eca-5HT1a transfected HEK293-GNA15 cells incubated with the indicated concentrations of serotonin, E; or serotonergic agonists, Sumatriptan, F; 8OH-DPAT, G and Quipazine, H.

Low affinity or very high concentration of the agonist sumatriptan (1000 µM) was necessary to detect a very slight increase in calcium levels (please compare calcium increase in F vs B). With the exception of serotonin (compare calcium increase in E vs A) which is a clear response, the three other agonists tested were considered as lacking activity in the cloned receptor.

The underlying causes of these technical issues are currently unclear but we are currently working trying to overcome them.

It could be hypothesized low levels of protein expression (or protein targeting in the membrane), however the heterologous system could have enough protein levels to detect activity with 5HT but not with other agonists.

Another hypothesis could be that this new receptor could have a different pharmacology (1).

It should be bearing in mind that no “selective” or “specific” drug exists that could bind only to one receptor but not to other types: most of the drugs binds to all receptors from all clades but with more “preference” for some types (2,3).

For the above reasons, we moderated the strength of the affirmations/conclusions related to receptor classification in the title, abstract and discussion sections. We delete the number “1” after 5HT from the title and short title. We employed the terms “likely”, “presumptive” and/or “putative” when we talk about receptor assignment to a specific clade. Please see lines 1, 2 and 4 in the titles, line 51 in the Abstract and lines 2083, 2085 and 2087 in the Discussion section in the “Revised Manuscript with Changes Highlighted” version.

**References**

1. Camicia F, Celentano AM, Johns ME, Chan JD, Maldonado L, Vaca H, Di Siervi N, Kamentezky L, Gamo AM, Ortega-Gutierrez S, Martin-Fontecha M, Davio C, Marchant JS, Rosenzvit MC. Unique pharmacological properties of serotoninergic G-protein coupled receptors from cestodes. PLoS Negl Trop Dis. 2018 Feb 9;12(2):e0006267. doi: 10.1371/journal.pntd.0006267
2. Tierney AJ. Invertebrate serotonin receptors: a molecular perspective on classification and pharmacology. J Exp Biol. 2018;221(Pt 19):jeb184838. Published 2018 Oct 4. doi:10.1242/jeb.184838
3. Tierney AJ. Structure and function of invertebrate 5-HT receptors: a review. Comp Biochem Physiol A Mol Integr Physiol. 2001;128(4):791‐804. doi:10.1016/s1095-6433(00)00320-2

Finally, there are some concerns around specificity of the serotonin antibody staining as part of the immunohistochemistry as staining seems non-specific in places. Inclusion or clarification around the application of a negative control for these methods is needed. Negative controls include exclusion of primary/secondary antibody.
Thank you for this interesting suggestion. We recognize important issues in serotonin staining. However, serotonin staining was already studied in previous works from the same species, see for example 4-7, in the references below (which were also included in the new version of the manuscript), for better and meticulous descriptions of the serotonergic neuroanatomy in the *Echinococcus* spp. In this work, we used serotonin staining only for comparative purposes to see where the new Eca-5HT1a receptor is located relative to the neurotransmitter serotonin which, according the results described, seems to be the ligand of this receptor. The serotonin localization or description *per se* was not the major aim of this manuscript.

Anyway, we removed the pictures with highest serotonin background staining (see for example, Fig 4), we also moderated the strength of the conclusions or affirmations derived by the use of this serotonin antibody in the text, we included one more control with excluded primary anti 5HT antibody (Fig 4, L) and finally discuss the results in the context of the current knowledge of the serotonergic neuroanatomy in *Echinococcus* spp. See lines 2174 to 2180.

**References**

4. Camicia F, Herz M, Prada LC, et al. The nervous and prenervous roles of serotonin in *Echinococcus* spp. Int J Parasitol. 2013;43(8):647‐659. doi:10.1016/j.ijpara.2013.03.006
5. Koziol U, Krohne G, Brehm K. Anatomy and development of the larval nervous system in *Echinococcus multilocularis*. Front Zool. 2013 May 4;10(1):24. doi: 10.1186/1742-9994-10-24
6. Brownlee DJ, Fairweather I, Johnston CF, Rogan MT. Immunocytochemical localization of serotonin (5-HT) in the nervous system of the hydatid organism, Echinococcus granulosus (Cestoda, Cyclophyllidea). Parasitology. 1994 Aug;109 (Pt 2): 233-41. doi: 10.1017/s0031182000076356.
7. Fairweather I, McMullan MT, Johnston CF, Rogan MT, Hanna RE. Serotoninergic and peptidergic nerve elements in the protoscolex of *Echinococcus granulosus* (Cestoda, Cyclophyllidea). Parasitol Res. 1994;80(8):649-56. doi: 10.1007/BF00932948

--------------------

**Results**

-Does the analysis presented match the analysis plan?
-Are the results clearly and completely presented?
-Are the figures (Tables, Images) of sufficient quality for clarity?

**Reviewer #1**: Results are clearly and appropriately presented.

**Reviewer #2:** The results are clearly represented. The figures need some adaptations (see below). Some results are not shown (see below).

**Reviewer #3:** The results presented are very detailed for 5-HT1aEchca and reflect the aims and hypotheses of the study. The presentation of *in silico* data and motif detail is extremely well done and easy to follow. This is information that is often difficult to find within the literature and the authors present a nice summary. Structural differences between receptor types is made very clear in the figures, however, the addition of receptor structure of a model organism (e.g. humans) in figure 5 would be good for comparison to show irrefutably that these receptors are structured in a similar way to type 1 and type 7 receptors in other organisms.
Thank you for this interesting suggestion. The figure was changed as suggested (now S3 Fig): the models of the human type 1 and 7 receptors were included in this figure to show that these receptors are structured in a similar way in other organisms.

The presentation of the heterologous expression data could be improved as graphs are visually messy and hard to read. They could be improved by distinguishing groups with colour or considering the statistical analysis of error bars presented. Changing the error bars to represent the standard error of the mean rather than standard deviation would reduce their size and make graphs easier to read.
Thank you for this interesting suggestion. The new Fig 3 was changed as suggested: colors were added in this version. Error bars were changed to represent the standard error of the mean as suggested.

Presentation of the localisation images could be improved. It is unclear what the difference between figure 8 and figure 9 is as they seem to present the same information. These figures could be combined and show a better representation of the overall expression patterns i.e. only show the best whole organism, anterior and posterior images. There is considerable non-specific staining of the serotonin antibody shown in  Figure 8B which should be removed, especially as the same information is presented better in figure 9D. All these images should be combined to make one figure if they are showing the same information as it is confusing to the reader.
Thank you for this interesting suggestion. Figures 7 and 9 were reorganized and included in one figure (Figure 4 in the new version). The figure 8B was removed and replaced by Figure 4 panel H, without serotonin staining, consistent with reviewer´s suggestions.

--------------------

**Conclusions**

-Are the conclusions supported by the data presented?
-Are the limitations of analysis clearly described?
-Do the authors discuss how these data can be helpful to advance our understanding of the topic under study?
-Is public health relevance addressed?

**Reviewer #1**: Conclusions are appropriate and supported by the data presented

**Reviewer #2:** The discussion and conclusions are supported by the data.

**Reviewer #3:** The authors conclude that they have identified a 5HT receptor that they believe to be type 1 and that responds to 5HT. They also state expression in the nervous system. These statements are valid based on the evidence presented.
The limitations around verifying the GPCR type based on heterologous expression are outlined, however, authors have not explained why they cannot use agonists/antagonists to the receptor as a way of verifying type.
Thank you for this interesting suggestion. We have performed some attempts to pharmacological profile Eca-5HT1a with some typical 5HT1 agonists in vertebrates like 8OH-DPAT, Quipazine and Sumatriptan. The results showed lack of activity of the agonists tested (see above). We are currently working to fix these technical problems.

There are some drugs considered as 5HT1 type specific based in the evidence presented mainly in serotonergic human receptors. However, we believe that this extrapolation is not always straightforward or valid and it could not be predicted that invertebrate receptors will behave the same pharmacologically as vertebrate receptors (8-10).

**References**

8. Camicia F, Celentano AM, Johns ME, Chan JD, Maldonado L, Vaca H, Di Siervi N, Kamentezky L, Gamo AM, Ortega-Gutierrez S, Martin-Fontecha M, Davio C, Marchant JS, Rosenzvit MC. Unique pharmacological properties of serotoninergic G-protein coupled receptors from cestodes. PLoS Negl Trop Dis. 2018 Feb 9;12(2):e0006267. doi: 10.1371/journal.pntd.0006267
9. Tierney AJ. Invertebrate serotonin receptors: a molecular perspective on classification and pharmacology. J Exp Biol. 2018;221(Pt 19):jeb184838. Published 2018 Oct 4. doi:10.1242/jeb.184838
10. Tierney AJ. Structure and function of invertebrate 5-HT receptors: a review. Comp Biochem Physiol A Mol Integr Physiol. 2001;128(4):791‐804. doi:10.1016/s1095-6433(00)00320-2

The authors present the data as identifying a potential novel drug target based on differences observed, however, this could be further enhanced with more information on how these receptors compare to human orthologues.
Thank you for this interesting suggestion. The receptors cloned here have interesting non-canonical residues at key positions like the DRY motif, PIF motif or toggle switch variations which are also different with their mammalian counterpart. This latter was further discussed in the discussion section. The hypothetical Eca-5HT1b seems to be more conserved at these residues with their mammalian orthologue, however, the cestode receptor has serine in position 5.46 not observed in other mammalian or invertebrate 5HT1 receptors with potential consequences at the pharmacological level.

Please see lines 2127 to 2133.

--------------------

**Summary and General Comments**

Use this section to provide overall comments, discuss strengths/weaknesses of the study, novelty, significance, general execution and scholarship. You may also include additional comments for the author, including concerns about dual publication, research ethics, or publication ethics. If requesting major revision, please articulate the new experiments that are needed.

**Reviewer #1:** This is a thorough, well performed and well-presented study of a new class of cestode serotonin receptor. The work has been carefully performed, with exceptional detail provided on receptor structural analysis. There is also reportage of technically challenging characterisation of receptor-ligand binding in a heterologous expression assay, and I welcomed the very nice confocal images of receptor and ligand co-localisation. I recommend acceptance of this MS with consideration of the following very minor changes:

> In discussion of receptor  N- and C-terminal domain lengths, the authors should consider stating that these have been perfomed on computationally-predicted sequences, and therefore may need to be biologically confirmed e.g. by targeted cloning and sequencing.
Thank you for this interesting input. We agree with this suggestion and indeed we expect some errors in sequences not confirmed by targeted cloning and sequencing. In the discussion we state that these predictions have been performed on computationally predicted sequences and therefore may need to be biologically confirmed as suggested. Please see lines 2094 to 2097.

> Naming convention is maybe a little unwieldy. Rather than 5-HT1aEchca (with subscript after the T), why not follow the "Eca-5HT1a" convention used in other work, which is simpler to my mind?
Thank you for this suggestion. We have changed all the receptor names in all the manuscript, including figures, tables and supplementary figures following the simpler naming convention suggested by this reviewer.

Line 56: insert "platyhelminth" before parasites
This was modified as suggested.

Line 106: Make clear whether the naming system used in this paper relates to mammalian or C. elegans 5-HT-R families
This was modified as suggested.

Line 120: "parasitical" should be changed to "parasitic"
This was modified as suggested.

**Reviewer #2:** In the manuscript by Camicia and colleagues, the authors investigate neurotransmitter signaling in cestodes, with a focus on the G protein coupled receptors (GPCRs) and serotonin signaling. They cloned putative serotonergic GPCRs of E. canadensis, M. vogae and H. microstoma, and bioinformatically analyzed and compared their sequences and structural predictions. The E. canadensis receptor was immunolocalized in the nervous system of protoscoleces (and beautiful immunostainings are shown), and also expressed in HEK cells. Here, the receptor could be stimulated with serotonin. The authors also elaborate on this receptor as a putative future drug target.

The entire study is clear, and the manuscript is very carefully prepared, with only few adjustments to be done.

The introduction appears comprehensive, and not too long.

One point that is missing from the results, is that the correct expression of the GPCR is shown (there were gels and blots made, as indicated in the methods, but the results are not shown). These results should not be given in text form only, and not in the methods (lines 938 ff).
The data of the ELISA is missing too.
Thank you for this interesting suggestion. We have included these results in the results section and designed a new supplementary figure which shows the results requested. The western blot results are now included in the S7 Fig, panel A and the ELISA results are included in the S7 Fig, panel B.

Why did the authors not apply a positive control for staining of the nervous system, and co-localize with the anti-GPCR antibody?
Thank you for this interesting suggestion. We agree with this proposal however we are unable to perform such studies for technical reasons: the antibodies available in the lab for staining of the nervous system (Anti Acetylated tubulin & anti synapsin) are monoclonal antibodies and the anti-GPCR antibody used in this work was produced also in mice. In summary, no double labelling is possible in this particular case. We would like to test other alternatives but we have extremely restricted local regulations to import new antibodies, high bureaucracy load and low budget to buy (and import) new antibodies. But anyway, we would be delighted to explore this possibility in the future.

Why did the authors not further assess the expression of the GPCRs in the disease-causing cyst stages by immunolocalization?
Thank you for this interesting suggestion. We only had access to protoscoleces (larval stage of the parasite) extracted from fresh livers which were from animals that were not specifically used for this study and all the material obtained was processed as part of the normal work of the abattoir. Cyst stages were not available during the course of this study but it would be very interesting to explore this possibility in the future.

The authors mention at the end of the discussion that inhibitors might be repurposed to be used against cestode GPCRs. Why did the authors not apply such already available drugs in their elegant system of GPCR activity by a fluorescent readout?
Thank you for this very interesting suggestion. Unfortunately, we were unable to detect calcium increases/ decreases using a discrete list of agonists different to serotonin. We only suggest that repurposing could be an interesting possibility to explore in the future.

The figure legends should be removed from the results section to the end of the manuscript.
Thank you for this suggestion. However, this suggestion seems to be incompatible with the PLOS ONE submission guidelines (https://journals.plos.org/plosone/s/submission-guidelines) in which it is clearly stated “Figure captions must be inserted in the text of the manuscript, immediately following the paragraph in which the figure is first cited (read order).”. We followed PLOS ONE submission guidelines and the figure legends were inserted in the text of the manuscript, immediately following the paragraph in which the figure is first cited.

Instead of long lists of protein sources in methods and figure legends, these could be given as a supplementary table. Like this, the figure legend 1 for example (lines 193-199) would much be shorter.
Thank you for this suggestion. We have designed Tables A, B and C in S1 Text with lists of receptor names, species and identification numbers which makes the text shorter.

The methods section is very detailed, and will allow for proper reproduction of the experiments.

Missing is the information on the codon-optimized sequence in line 917. The sequence should be given (in the supplementaries).
Thank you for this suggestion. The original and optimized nucleotide sequences together with the predicted amino acid sequence is now provided in the new version of the manuscript (now S2 Text).

For the figures, figures 1 and 2 be unified in one figure with subfigures. Also figures 3-5 could be part of one figure with subfigures. Figure 5 could even be shown as supplementaries only. Figures 7-9 could also be shown as one figure with subfigures.
Thank you for this suggestion. Figures 1 and 2 were unified in one figure with subfigures as suggested. Figures 3 and 4 were also combined in one figure. Figure 5 was changed to S3 Fig and shown as supplementary. Figures 7, 8 and 9 were combined and shown as one figure with subfigures. In summary, all the changes were made as suggested.

In Figure 6 (and also respective supplementary figures), the legend title is missing.

Thank you for this suggestion. All the figure legends titles were shortened and re-written in a more concise way according to PLOS ONE guidelines.

Please use thinner lines and/or grey colors for the SDs, so the data points are well visible.
Thank you for this suggestion. This figure (now Figure 3), was modified as suggested. Thinner lines were used and grey colors for the s.e.m.

For Figure 4, next to the overview structures on the top, indicate “domain III” on the left side, and “domains V and VII” on the right side of the figure.
Thank you for this suggestion. This Figure (now Figure 2) was modified and clarified as suggested with clear and concise vertical titles in the left side of the figures.

**Reviewer #3:** The authors aim to describe the characterisation of a novel type 1 5HT receptor expressed in *Echinococcus canadensis* with homologues in closely related species. This work is novel such that no other literature exists to functionally characterise type 1 5HT receptors in platyhelminthes. The receptor is presented as having key differences to other 5HT GPCRs which could have significant implications for drug target development.

I recommend that this manuscript is accepted with revision. The authors have done an outstanding job of outlining the *in silico* analysis for 5-HT1aEchca, however, have neglected to give the same attention to 5-HT1bEchca, which they also describe as a novel type 1 receptor. The greatest strength of this manuscript is the detail that the authors provide on conserved motifs and 5HT GPCR structure which is often difficult to find and collate in the literature. This manuscript provides a very good summary of 5HT receptor conserved domains and it would be great to see this expanded to include more information on 5-HT1bEchca.
Thank you very much for this comment. We have included more information on Eca-5HT1b: multiple sequence alignment of this sequence (S2 Fig), ligand binding and coupling sites (S2 Fig; S4 Fig), coupling score (new Table 4), transmembrane domains (S2 Fig), molecular modelling studies and length of the ICL3 and C-terminal end (S4 Fig) are now provided in this new version of the manuscript.

Weaknesses are obvious when presenting data on the functional characterisation of 5-HT1aEchca. The authors have neglected to include simple experiments using 5HT receptor agonists/antagonists during heterologous expression investigations to irrefutably prove that this receptor is in fact specific as a serotonin GPCR and is type 1 in its grouping. As there are key differences in the amino acid sequence that make this receptor unusual when compared to other serotonin GPCRs, further functional studies are essential to validate the *in silico* data.

Thank you very much for this suggestion. Unfortunately, further attempts to characterize this receptor by further pharmacological profiling were unsuccessful. We have only proven that this receptor is activated by serotonin. The bioinformatics analyses only suggest that this receptor is of type 1 but we are currently unable to irrefutably prove that this receptor is in fact of type 1. For these reasons, we have tempered our assertions about assignment of this receptor in the titles, summary and also in the discussion sections as a type 1. Please see 1, 2 and 4 in the titles, line 51 in the Abstract and lines 2083, 2085 and 2087 in the Discussion section.

The antibody staining for 5-HT1aEchca is really good and shows clear and specific expression of this receptor in neuronal tissues as the authors outline. However, the comparison of receptor expression with serotonin protein expression is not very clear. The serotonin staining in a lot of places is non-specific and this is especially obvious around the anterior end and tegument. The overall staining is poor and it seems that where there is good receptor staining there is poor serotonin staining and vice versa (9A+9B) which makes it difficult to make definitive conclusions. The authors state that expression patterns of receptor and serotonin are considerably different in places, however, it is possible that patterns are masked by poor serotonin staining. Authors should consider tweaking the IHC protocol and attempting to improve these images before drawing conclusions or state difficulties in obtaining clear serotonin staining.

Thank you very much for this suggestion. We agree and recognize some issues in serotonin staining probably related to the low titer of the anti-serotonin antibody. For this reason, we were forced to use this antibody at very high concentrations/low dilutions (dilution of 1 in 50, when the usual dilution is of 1 in 1000) in order to clearly see neuronal ramifications and ganglia staining, which probably results in a non-specific staining, especially in the tegument. Unfortunately, we are currently unable to repeat these IHCs due to the restricted availability of the parasitic material. We are also unable to get fresh anti-serotonin antibody.

We stated some difficulties in obtaining clear serotonin staining and moderated the strength of the affirmations/conclusions related to serotonin staining in the discussion section. See lines 2174 to 2180.

Overall, I believe this publication contains important information that should be disseminated to the community, however, further information is needed as outlined to do so in a more detailed and definitive manner.

--------------------

PLOS authors have the option to publish the peer review history of their article (<https://journals.plos.org/plosntds/s/editorial-and-peer-review-process#loc-peer-review-history> what does this mean?). If published, this will include your full peer review and any attached files.

If you choose “no”, your identity will remain anonymous but your review may still be made public.

Do you want your identity to be public for this peer review?

For information about this choice, including consent withdrawal, please see our <https://www.plos.org/privacy-policy> Privacy Policy.

**Reviewer #1:** No

**Reviewer #2:** No

**Reviewer #3:** No

--------------------

**Editorial and Data Presentation Modifications?**

Use this section for editorial suggestions as well as relatively minor modifications of existing data that would enhance clarity. If the only modifications needed are minor and/or editorial, you may wish to recommend “Minor Revision” or “Accept”.

**Reviewer #2:** The language is well-written and clear. The authors should use the abbreviations consistently for “5-HT7” and for “5-HT2” (sometimes “5HT2” or “5HT7”). Instead of C57 mice, the term C57BL/6 should be used. Consistently use either “s.s.” and “s.l.” or “sensu stricto” and “sensu lato”
Thank you for this suggestion. The abbreviations were modified as suggested. The terms “sensu stricto” and “sensu lato” were used when first named in the text, then, the abbreviated forms “s.s.” or “s.l.” were used.

In the section from line 272-282, amino acids are sometimes abbreviated by the single letter code, albeit throughout the manuscript, full amino acid names are given. This should be handled consistently in one or the other way.
Thank you for this suggestion. In the section referred, amino acids were named using full amino acid names as suggested.

Abstract/Introduction
-       Lines 35 and 73: syn not capitalized
This was modified as suggested.

Results
-       Line 398 and following: call it “loop III” as in the figure
This was modified as suggested.

Figure legend 1
-       Line 180: “… is shaded in grey.”
-       Line 183: “… names are marked…”
-       Line 184: “… domains are indicated…”
-       Line 188: “… are indicated…”
-       Line 189: “… are underlined.”
-       Line 191: “… formation are…”
All this was modified as suggested.

Figure legend 2
-       Line 210: “… with a repertoire…”
-       The scale bar mentioned in line 221 is missing in the figure
All this was modified as suggested. The scale bar is now included in the new Figure 1, panel C.

Figure legend 3
-       Line 348: is PDB4IAR the human receptor? Please write more clear
-       Line 354: “… are represented…”
All this was modified and clarified as suggested.

Figure legend 4
-       Line 394: “… structures are represented…”
-       Line 395: “… involved are…”
All this was modified as suggested.

Figure legend 5
-       Line 405: “… are represented as cartoons…”
-       Line 406: “… are represented…”
-       Line 409: “… which a bigger…”
All this was modified as suggested.

Figure legend 6
-       Line 430: space between 10 uM
-       Line 432: “… are:….”
-       Indicate also how many repetitions or technical replica are shown in the mean data
All this was modified as suggested.

Figure legend 7
-       Indicate the colors for the different antibodies in the first sentences instead of several times repeatedly for each subfigure
-       Line 465: “… protoscolex shown…”
-       Line 469: “… shown…”
-       Line 475: “… which were later…”
-       Line 477: “… are marked…”
All this was modified as suggested.

Figure legend 8
-       Line 488: “C and D”
This was modified as suggested.

Figure legend 9
-       Indicate the colors for the different antibodies in the first sentences instead of several times repeatedly for each subfigure
-       Line 502: “shown”
-       Line 505: “shown”
-       Line 508: “… scale bar is…”
All this was modified as suggested.

Discussion
-       Line 566: “Possibly, these…”
-       Line 597: “… same as those…”
All this was modified as suggested.

Methods
-       Line 686: only the stock concentration of 5-HT is indicated. What were the stock solutions for the other chemicals tested?
Thank you for this interesting suggestion. The stock solutions for the other chemical are provided as suggested in the text.

-       The accession numbers from line 737-748 should be given in a supplementary table
Thank you for this interesting suggestion. We have removed all the accession numbers from these lines and transferred to Tables in S1 Text as suggested.

-       Line 844: “H. microstoma”
-       Line 897: “in situ” in italics
-       Line 919: “E. coli” and in italics
-       Line 966: species should be indicated for the protoscoleces
-       Line 984: “glycerol”
All this was modified as suggested.

**Reviewer #3**: Overall this manuscript is written and presented very well.

Minor changes to text recommended as follows;

Line 158 – unclear whether discussing receptor A or B
Receptor A was specified here as suggested.

Line 221 – no scale bar obvious on figure
Scale bar was included as suggested.

Line 375 – spelling mistake, should read ‘alpha’
This mistake was corrected as suggested.

Line 393-395 – unclear the meaning of ‘sticks’
The meaning of “sticks” was clarified in the new figure legend.

Line 428 & 429 – wrong legend points in brackets; solid circles are closed squares and open circles are closed circles
This was modified as suggested.

Line 602 – minor staining reads subjective; there is quite a lot of staining

Thank you for this suggestion. We have clarified this point in the text stating that minor staining refers to Eca-5HT1a and not to serotonin staining.
